# Proteogenomic Characterization Reveals Metabolic Vulnerabilities and Aberrant Phosphorylation in Colorectal Metastasis to Liver

**DOI:** 10.1002/advs.202511744

**Published:** 2025-11-06

**Authors:** Wensi Zhao, Lei Zhao, Yannan Lian, Zhiwei Liu, Yaqi Li, Xuege Wang, Mingya Zhang, Ni Li, Jingli Guo, Danqing Shen, Shaobo Mo, Jiahao Li, Linhui Zhai, Jiahui Ni, Sangkyu Lee, Bin Liu, Jing Li, Fei Wang, Junjie Peng, Jun Qin, Minjia Tan

**Affiliations:** ^1^ Translational Research Institute of Brain and Brain‐Like Intelligence Shanghai Fourth People's Hospital, and Cancer Center School of Medicine Tongji University Shanghai 200434 China; ^2^ Shanghai Institute of Nutrition and Health Chinese Academy of Sciences 320 Yueyang Road Shanghai 200031 China; ^3^ State Key Laboratory of Drug Research Shanghai Institute of Materia Medica Chinese Academy of Sciences Shanghai 201203 China; ^4^ Department of Colorectal Surgery Fudan University Shanghai Cancer Center Department of Oncology Shanghai Medical College Fudan University Shanghai 200032 China; ^5^ Department of Bioinformatics and Biostatistics School of Life Sciences and Biotechnology Shanghai Jiao Tong University Shanghai 200240 China; ^6^ Shanghai Key Laboratory of Intelligent Information Processing School of Computer Science and Technology Fudan University Shanghai 200433 China; ^7^ School of Pharmacy Sungkyunkwan University Suwon 16419 Republic of Korea; ^8^ Jiangsu Key Laboratory of Marine Pharmaceutical Compound Screening College of Pharmacy Jiangsu Ocean University Lianyungang 222005 China; ^9^ Jinfeng Laboratory Chongqing 401329 China; ^10^ Zhongshan Institute for Drug Discovery, Shanghai Institute of Materia Medica Chinese Academy of Sciences Zhongshan 528400 China

**Keywords:** biomarker, colorectal liver metastasis, phosphoproteomics, proteomic subtype, proteomics, SHMT1

## Abstract

Colorectal liver metastasis (CRLM) is one of the leading death causes among colorectal cancer (CRC) patients, yet its underlying molecular events remain poorly understood, particularly at the proteomic and phosphoproteomic levels. A proteogenomic analysis combining genomics, transcriptomics, proteomics, and phosphoproteomics is performed on 102 samples from 34 treatment‐naïve CRLM patients, including primary CRC, adjacent normal colorectal, and matched liver metastasis tissues. CRC cell lines, organoids, mouse models, and an independent patient cohort are used to validate the findings. Proteomics and phosphoproteomics show profoundly dysregulated pathways in liver metastasis tissues, notably disruptions in carbon metabolism. Functional validation using CRC organoids and mouse models demonstrates that the one‐carbon metabolism enzyme SHMT1 promotes CRC tumorigenesis and metastasis via formate‐mediated AMPK inhibition, whereas PIM kinase‐dependent NDRG1 Ser330 phosphorylation exacerbates liver metastasis by promoting ubiquitin‐dependent degradation of NDRG1. Unsupervised clustering identifies two proteomic subtypes of liver metastasis samples with distinct clinical outcomes: a poor‐prognosis C1 (metabolism) subtype and a better‐prognosis C2 (RNA function) subtype. Considering expression frequency, specificity, and functional relevance, FTCD, GPD1, SOD2, and EIF4B Ser422 phosphorylation are further identified and validated as subtype prognostic biomarkers. This study provides critical insights into the molecular mechanisms underlying CRLM and offers resources for high‐risk metastatic CRC.

## Graphical abstract

This study provides a multi‐omics landscape of treatment‐naïve colorectal liver metastasis and reveals dysregulated molecules and cellular pathways. SHMT1 and NDRG1 Ser330 phosphorylation are demonstrated to display crucial roles in tumorigenesis and liver metastasis. Two proteomics subtypes (metabolism and RNA function) with distinct clinical outcomes and potential prognostic biomarkers are identified.

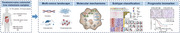

## 1 Introduction

Colorectal cancer (CRC) is the third most common cancer worldwide, with a high cancer‐related death rate and ≈14% of 5‐year survival rate.^[^ ^1^, ^2^, ^3^ ^]^ The liver is the most frequent CRC metastasis site due to the rich blood supply and its anatomical situation. Nearly 50% of the CRC patients develop liver metastasis during the disease progression, which always leads to poor prognosis.^[^ ^4^, ^5^ ^]^ Surgical resection of colorectal liver metastasis (CRLM) is recognized as the primary treatment that offers the possibility of cure. The anatomical location of the primary tumor, the histological growth patterns of liver metastasis tumor, and the presence of *RAS* mutations have been identified as factors contributing to distinct clinical outcomes.^[^ ^6^, ^7^, ^8^, ^9^, ^10^ ^]^ Accordingly, the clinical risk score (CRS) was proposed to predict the prognosis after hepatic resection for metastatic CRC (mCRC).^[^ ^11^ ^]^ However, its use has shown limitations in forecasting prognosis and guiding treatment improvements in clinical practice.^[^ ^12^ ^]^

Various molecular and signaling pathway alterations contribute to the occurrence and progression of CRLM.^[^ ^13^ ^]^ As a key metabolic organ, the liver's metabolic pathways are often hijacked by metastatic tumors to facilitate their outgrowth.^[^ ^14^, ^15^ ^]^ Outside the cancer cells, the tumor microenvironment (TME) of liver metastasis harbors a highly immunosuppressive phenotype, leading to a systemic loss of antigen‐specific T lymphocytes and enhanced invasiveness and metastasis of primary cancer cells.^[^ ^13^, ^16^ ^]^ Consequently, immunotherapy showed limited success for patients with CRLM. Therefore, more research is needed to uncover the molecular mechanism underlying CRLM.

Molecular profiling of CRC tumors based on genomics technologies has shown the improvement of overall survival (OS) in certain patient subtypes.^[^ ^17^ ^]^ Nevertheless, recent studies have revealed that the genomic differences between the primary tumor and liver metastasis are relatively slight, suggesting that genomic alterations are unlikely to be the primary drivers of CRLM.^[^ ^18^, ^19^ ^]^ Notably, studies primarily relied on genomic‐level analysis, which focuses on specific biological layers of CRLM, and failed to fully elucidate the complexities of molecular profiles and might thus overlook crucial molecular characteristics. Since proteins are the final gene products responsible for cellular functions,^[^ ^20^, ^21^ ^]^ an in‐depth characterization of the proteomics (protein expression) and phosphoproteomics (signal transduction) would facilitate a more comprehensive understanding of CRLM‐related biology, laying a stronger foundation for the development of precision medicine. To date, the proteomic and phosphoproteomic characteristics critical for CRLM are still largely unclear.

Recent proteomic and phosphoproteomic characterization of primary CRC tissues provided unique functional insights complemented by the genomic information.^[^ ^22^, ^23^, ^24^, ^25^ ^]^ The first large‐scale proteomic characterization of primary CRC tissues by Zhang et al. provided new functional insights, complemented with the genomic technology‐driven studies and practice of CRC.^[^ ^22^ ^]^ Their following proteomic profiling study of 44 CRC cell lines showed evidence of specific drug sensitivity for proteomics‐based CRC subtypes.^[^ ^23^ ^]^ Their subsequent integrative proteogenomic and phosphoproteomic study of 110 primary colon cancer tissues disclosed that the retinoblastoma protein (Rb) phosphorylation was associated with colon cancer development, and glycolysis inhibition showed potential in microsatellite instability‐high (MSI‐H) subtype tumors.^[^ ^24^ ^]^ A recent study carried out an integrated multi‐omics analysis of trace CRC samples with different stages to delineate driving events in the progression stage.^[^ ^25^ ^]^ However, these proteogenomic studies focused on primary CRC rather than mCRC liver samples. Li et al. carried out multi‐omics analysis of mCRC patients and illustrated the profound heterogeneity between primary tumor and liver metastasis at the proteomic and phosphoproteomic levels, which indicated significant differences in the kinase‐phosphosubstrate networks and drug responses between primary tumor and liver metastasis.^[^ ^26^ ^]^ Nonetheless, this study included both pre‐therapy (chemotherapy or targeted agents) and treatment‐naïve CRC samples. Since drug treatment inevitably alters the intrinsic proteomic characteristics and TME of tissue samples, this study could not provide a comprehensive molecular profile reflecting the original pathological and biological features of CRLM. Additionally, the molecular heterogeneity and signaling pathways within liver metastases, as well as their associations with clinical features and outcomes, remain inadequately explored to date.

In this study, we collected 102 clinical samples from 34 CRLM patients, including CRC tissues (T), matched liver metastasis tissues (LM), and adjacent normal colorectal tissues (N). We established a comprehensive multi‐omics landscape, including proteomics, phosphoproteomics, genomics, and transcriptomics. A large number of cellular pathways were dysregulated between LM and T samples at the proteomic and phosphoproteomic levels, notably those involved in carbon metabolism, actin cytoskeleton dynamics, complement regulation, and RNA splicing. Importantly, serine hydroxymethyltransferase 1 (SHMT1) in the carbon metabolism pathway, and the metastasis suppressor N‐myc downstream‐regulated gene 1 (NDRG1) Ser330 phosphorylation, were identified to display crucial roles in tumorigenesis and liver metastasis. Two proteomics subtypes (metabolism and RNA function) with distinct clinical outcomes and potential prognostic biomarkers were identified.

## 2 Results

### 2.1 Multi‐Omics Landscape of Primary CRC and Liver Metastasis

**Figure 1:**
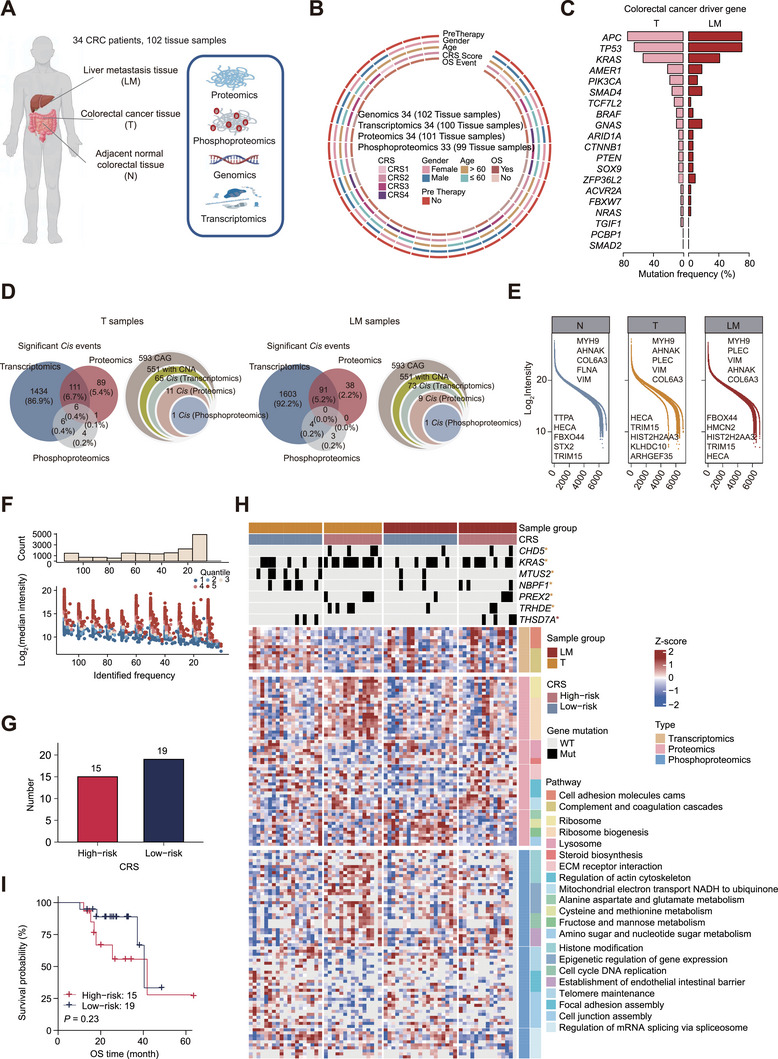
Multi‐omics characterization of CRLM samples. A) Scheme of the genomic, transcriptomic, proteomic, and phosphoproteomic analyses of CRLM clinical samples. B) Patient clinical information and the number of tissue samples used for genomic, transcriptomic, proteomic, and phosphoproteomic analysis, respectively. C) Mutation frequency of CRC driver genes in primary tumor and liver metastasis samples. D) Venn diagram summarizing the significant *cis* effects of all the SCNAs at the transcriptomics, proteomics, and phosphoproteomics levels (Spearman correlation, FDR < 0.05) in T (left) and LM (right) samples. The overlap of the significant *cis* events of 593 cancer‐associated genes at the transcriptomics, proteomics, and phosphoproteomics levels (Spearman correlation, FDR < 0.05) in T (left) and LM (right). E) Distribution of protein intensity with median normalization and log_2_ transformation. The top five highest and lowest abundant proteins were listed in N, T, and LM samples, respectively. F) Distribution of phosphosite intensity with different identified frequencies. The median intensity of phosphorylation sites across all samples was transformed by log2, and the phosphosites in each identified frequency group were colored according to the quantile of the intensity. In the upper panel, the bar plot showed the count of phosphorylation site. G) The number of CRLM patients with high (CRS ≥ 3) and low (CRS < 3) CRS. H) Heat map summarizing gene mutation, transcription expression, protein expression, and phosphorylation site abundance between high and low CRS groups in T and LM samples. Clinical samples are grouped based on high or low CRS. Differential mRNAs, proteins, and phosphosites were filtered with *p* < 0.05 and fold‐change ratios of 1.5, 1.2, and 1.5, respectively (two‐sided Wilcoxon rank sum and signed rank tests). Their associated biological processes and KEGG pathways were labeled on the right (FDR < 0.05). I) Kaplan‐Meier curves of overall survival (OS) in patients with high and low CRS (log‐rank test, *n* = 34). ^∗^ represented *p* < 0.05.

We collected a cohort of 102 CRLM clinical samples, including 34 T samples with matched N and LM samples for genomics, transcriptomics, proteomics, and phosphoproteomics analyses (**Figure** **1**A,B; Figure S1A and Table S1, Supporting Information). In total, 19493 nonsynonymous somatic mutations and 23109 somatic copy‐number alterations (SCNAs) were identified from whole exome sequencing (WES) analysis (Table S2, Supporting Information). The mutation frequencies of CRC typical driver genes, including *APC*, *TP53*, and *KRAS*, showed no obvious difference between paired T and LM samples (Figure 1C; Figure S1B, Supporting Information), which is similar to a prior report.^[^ ^19^ ^]^ The significant amplifications shared by paired T and LM samples were on chromosomes 1q, 13q, 15q, and 17q, and deletions were on 1p, 10q, 12q, and 17q (FDR < 0.05). LM samples uniquely identified amplification on chromosome arm 8p and deletions on chromosome arms 8p, 12q, and 16p, whereas T samples uniquely identified amplifications on arms 17p and 20q and deletions on arms 1q, 2p, 2q, 5q, 7q, 14q, 17p, 21q, and 22q (FDR < 0.05). To explore the impact of SCNAs on mRNA, protein, and phosphoprotein abundance, we performed the correlation analysis (Figure 1D), and the results showed that cis‐regulatory effects of SCNA on mRNAs were more prominent than those of proteins or phosphoproteins. According to a well‐defined dataset of cancer‐associated genes (CAG) to nominate functionally important genes with cis‐regulatory effects,^[^ ^26^, ^27^ ^]^ 551 genes were identified in our cohort, including *PDS5B*, which showed *cis* effects at multiple omics levels in both T and LM (Figure 1D).

Tandem mass tag (TMT) labeling approach was used for proteomic and phosphoproteomic quantification using high‐resolution mass spectrometry (MS). Proteomics analysis identified 8568 proteins and quantified 8093 proteins (Figure 1E; Figure S1C; Table S2, Supporting Information). Ti^4+^‐IMAC‐based phosphoproteomic analysis identified 25775 phosphosites and quantified 19803 phosphosites, including 16300 class I phosphosites and 3503 class II phosphosites (Figure 1F; Figure S1C,D and Table S2, Supporting Information). Class I phosphosites were used for further analysis. Correlation analysis of internal reference sample (IRS) in proteomics and phosphoproteomics demonstrated the platform stability, reproducibility, and reliability (Figure S1E, Supporting Information), and quality control showed good quality for all proteomic and phosphoproteomic samples (Figure S1F, Supporting Information). A gene‐wise mRNA‐protein correlation analysis revealed a strong overall positive association (*p* < 0.05, ρ > 0.3), with only a minimal set of genes showing negative regulation (*p* < 0.05, ρ < −0.3) (Figure S1G, Supporting Information). Gene ontology (GO) biological process (BP) enrichment analysis showed that the positively correlated genes were enriched with metabolic processes, such as ribonucleotide and fatty acid metabolic processes, highlighting the complex global transcription and protein activity regulatory networks in CRC. Principal component analysis (PCA) on multi‐omics data revealed that proteomics data exhibited the strongest discriminative power among transcriptomic, proteomic, and phosphoproteomic profiles in distinguishing N, T, and LM samples. (Figure S1H, Supporting Information).

CRS takes five factors into consideration to predict the prognosis after hepatic resection for mCRC,^[^ ^11^ ^]^ and high CRS (CRS ≥ 3, high‐risk group) is associated with poor prognosis. We next applied the CRS criteria to our CRLM patients and explored the molecular characteristics (Figure 1G). Our multi‐omics data showed that T samples with high CRS were closely related to ribosome, epigenetic regulation, histone modification, and establishment of the endothelial intestinal barrier, whereas T samples with low CRS (CRS < 3) were enriched with cell adhesion, lysosome, and steroid biosynthesis (Figure 1H). In LM samples, the high CRS group was associated with ECM receptor interaction, regulation of actin cytoskeleton, and mitochondrial electron transport NADH to ubiquinone, while the low CRS group was enriched with amino acid metabolism, fructose and mannose metabolism, and mRNA splicing events. Nevertheless, high CRS was not significantly (5‐year OS, log‐rank *p* = 0.23) correlated with poor prognosis in our mCRC patients (Figure 1I; Figure S1I, Supporting Information), and immune analysis showed barely differentially expressed immune‐related molecules (Figure S1J, Supporting Information), suggesting the limitation of CRS for prognostic prediction.

### 2.2 Comprehensive Multi‐Omics Characterization of Primary CRC and Liver Metastasis

**Figure 2:**
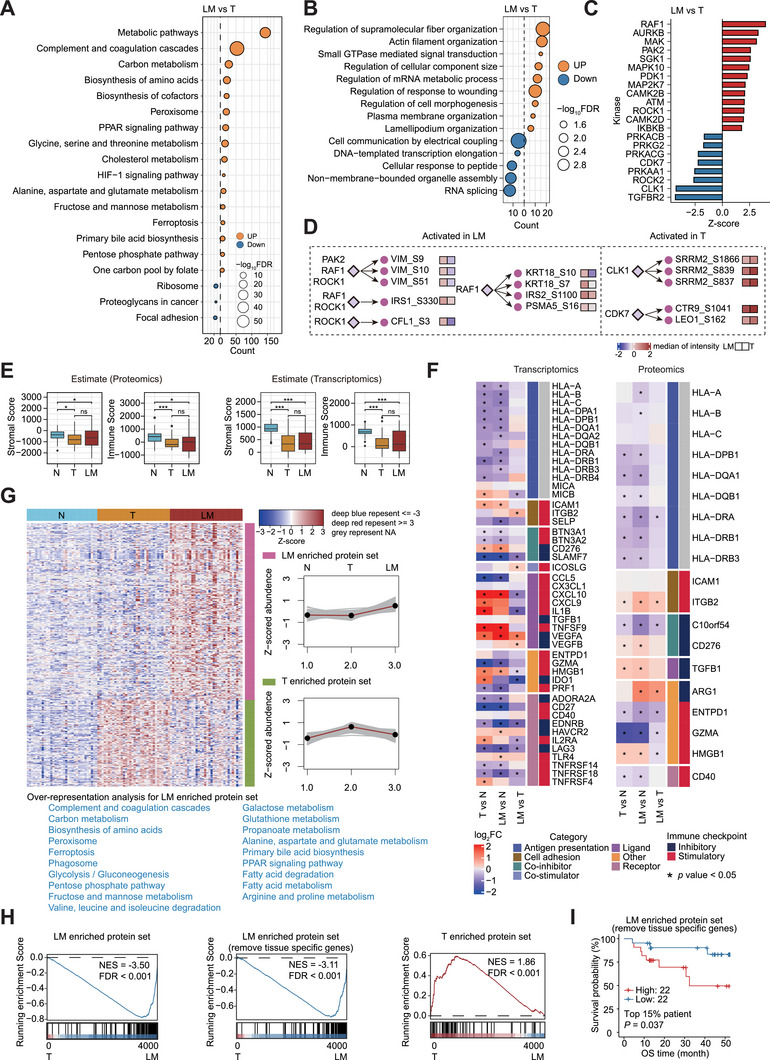
Comprehensive characterization of the primary CRC and liver metastasis samples. A) Representative KEGG pathways enriched in T and LM based on proteomics data (FDR < 0.05). B) Representative biological processes enriched in T and LM based on phosphoproteomics data (FDR < 0.05). C) Kinase activities in LM (red) and T (blue) samples were evaluated by KSEA (*p* < 0.05). D) Representative kinase‐substrate relationships were accordingly annotated. E) Boxplot of immune scores and stromal scores at proteomic level and transcriptomic level for N, T, and LM samples, respectively (two‐sided Wilcoxon rank sum and signed rank tests). F) Heat map presenting mRNA and protein expression of immunomodulatory genes (two‐sided Wilcoxon Rank Sum and Signed Rank Tests).^[^ ^36^ ^]^ G) Heatmap of protein expression of LM‐ and T‐enriched protein sets. KEGG pathway enrichment analysis (FDR < 0.05) of the LM‐enriched protein set. H) Gene set enrichment analysis using a publicly available proteomic dataset (Li et al. dataset^[^ ^40^ ^]^). The LM‐enriched protein set with and without tissue‐specific genes, and the T‐enriched protein set were utilized as the molecular signature database. I) Kaplan‐Meier curve of OS in patients from the Li et al. dataset (log‐rank test). The protein expression was calculated as the median of the LM‐enriched protein set without tissue‐specific genes for each patient. ^∗^ represented *p* < 0.05, ^∗∗^ represented *p* < 0.01, ^∗∗∗^ represented *p* < 0.001 and ns represented not significant.

To systematically identify the functional molecules and events between primary CRC and liver metastasis, we first conducted comparative analysis among N, T, and matched LM samples at the proteome level (**Figure** **2**A; Figure S2A and Table S3, Supporting Information). Compared with LM samples, overexpressed proteins in T samples were enriched in focal adhesion pathway (FDR < 0.05). In contrast, LM samples showed the upregulated proteins associated with complement and coagulation cascades, PPAR signaling, and diverse metabolic pathways such as carbon metabolism, cholesterol metabolism, fructose and mannose metabolism, and amino acid metabolism (FDR < 0.05). The enrichment in multiple metabolism pathways suggested that elevated metabolism activity in the liver may facilitate CRC cells to survive and adapt to the new hepatic environment.

Protein phosphorylation plays a prominent role in cancer initiation and development.^[^ ^28^, ^29^, ^30^ ^]^ GO BP enrichment analysis was performed on differentially expressed phosphosites (fold change > 1.5 and Wilcoxon rank sum and signed rank tests, *p* < 0.05) among N, T, and matched LM samples (Figure 2B; Figure S2B and Table S3, Supporting Information). Compared with T samples, LM samples significantly affected phosphorylation events in actin filament organization, small GTPase‐mediated signal transduction, regulation of cell morphogenesis, and lamellipodium organization (FDR < 0.05). In contrast, the cell communication process by electrical coupling, cellular response to peptide, and RNA splicing were significantly enriched in T samples, indicating different underlying mechanisms between liver metastasis and primary tumors. RNA splicing dysregulation plays a pervasive and causative role in cancer initiation and progression.^[^ ^31^, ^32^ ^]^ To identify the distinct alternative splicing (AS) events between LM and T samples, we performed rMATS analysis based on the RNA‐seq data.^[^ ^33^ ^]^ In total, we identified 288337 AS events in LM and T samples, including 12185 alternative 3′ splice sites (A3SS), 7985 alternative 5′ splice sites (A5SS), 51671 mutually exclusive exons (MEX), 5537 retained introns (RI), and 210959 skipped exons (SE). Among them, 26 AS events showed a significant difference between LM and T samples at a threshold of FDR < 0.05 and absolute IncLevelDifference > 0.1. Survival analysis revealed that 14 out of 26 AS events were correlated with prognosis (Figure S2C, Supporting Information). *FN1*, *KHK*, and *SERPINA1* were the top three genes with the highest number of significantly different AS events, which were all associated with OS (Figure S2D, Supporting Information). In addition, the protein expression levels of two spliceosome‐related factors, DDX5 and SF1, were significantly downregulated in LM samples and were associated with prognosis (Figure S2E, Supporting Information). Kinase substrate enrichment analysis (KSEA) showed multiple kinases, including RAF1, PAK2, and ROCK1, were hyperactivated in LM samples, while the kinase activities of CLK1, CDK7, and PRKACB were upregulated in T samples (Figure 2C). As can be seen in Figure 2D, RAF1, PAK2, and ROCK1 showed the potential to phosphorylate vimentin (VIM), a key epithelial‐mesenchymal transition (EMT) marker in cell invasion and metastasis.^[^ ^34^ ^]^ Moreover, ROCK1 was predicted to affect the Ser3 phosphorylation of Cofilin‐1 (CFL1), which also participated in the EMT process.^[^ ^35^ ^]^

To depict the immune and microenvironment signatures between T and LM samples, we analyzed the immune score, stromal score, the distribution of immune cell types, and the expression of immunomodulatory genes using ESTIMATE (Estimation of STromal and Immune cells in MAlignant Tumor tissues via Expression data), xCell, and the immune database,^[^ ^36^, ^37^ ^]^ respectively. Generally, both T and LM samples presented relatively lower immune scores and stromal scores compared to N samples (Figure 2E). According to xCell analysis, M2 macrophages were significantly higher in LM samples than in T samples (Figure S2F, Supporting Information). A number of immunomodulatory genes with the function of antigen presentation were significantly down‐regulated in primary tumor and liver metastasis at transcriptomic, proteomic, or phosphoproteomic levels (Figure 2F; Figure S2G, Supporting Information). Interestingly, Arginase‑1 (ARG1), an immune suppressor, was conspicuously overexpressed in liver metastasis on the protein level rather than the mRNA level (Figure 2F; Figure S2H, Supporting Information). ARG1 was classified as a plasma protein, and the high expression of ARG1 correlated with poor prognosis in T and LM samples (Figure S2I, Supporting Information), suggesting a potential role in CRC liver metastasis.

### 2.3 Proteomics Identifies the Molecular Signatures of CRLM

To distinguish the molecular signatures between liver metastasis and primary tumor, we calculated differentially expressed proteins to identify the LM‐ or T‐enriched protein sets (Table S4 (Supporting Information), detailed method in Supporting Information, LM‐ or T‐enriched protein set selection and GSEA analysis section). Consistent with the previous studies that showed CRC cells underwent metabolic reprogramming to adapt the liver environment,^[^ ^15^, ^38^, ^39^ ^]^ LM‐enriched protein set (*n* = 245) predominantly contained proteins with metabolism relevance, and were closely related with a series of metabolic pathways, such as fructose and mannose metabolism, pentose phosphate pathway, carbon metabolism, and amino acid metabolism pathways (Figure 2G). To further evaluate the power of these two LM‐ or T‐enriched protein sets, we performed gene set enrichment analysis (GSEA) for the only publicly available CRC liver metastasis proteomics data (Li et al. dataset^[^ ^40^ ^]^) using our defined T‐ and LM‐enriched protein sets as the molecular signature database. The results showed LM samples from Li et al. dataset were significantly enriched (FDR < 0.001, NES = −3.50) with our LM‐enriched protein set, whereas the T samples from Li et al. dataset were closely related with our T‐enriched protein set (FDR < 0.001, NES = 1.86) (Figure 2H). To rule out tissue‐specific effects, we excluded tissue‐specific genes, and results were similar (Figure 2H, FDR < 0.001, NES = −3.11), confirming the capacity of our LM protein set to distinguish primary tumors from liver metastases. Further prognostic analysis using the median protein expression of the LM‐enriched protein set in the T samples (*n* = 146) of the Li et al. dataset showed that high protein expression level of LM‐enriched proteins was significantly correlated (log‐rank *p* = 0.037) with a poor OS rate (Figure 2I).

To facilitate the translational applications, we further narrowed down the protein list with more filter criteria (Table S4, Supporting Information for more details), which resulted in 96 proteins in the T‐enriched protein set and 200 proteins in the LM‐enriched protein set (155 proteins after removing tissue‐specific genes), and similar results were obtained (Figure S2J,K, Supporting Information).

### 2.4 Proteomics Reveales SHMT1 Promotes CRC Tumorigenesis and Metastasis via Formate‐Mediated AMPK Inhibition

**Figure 3:**
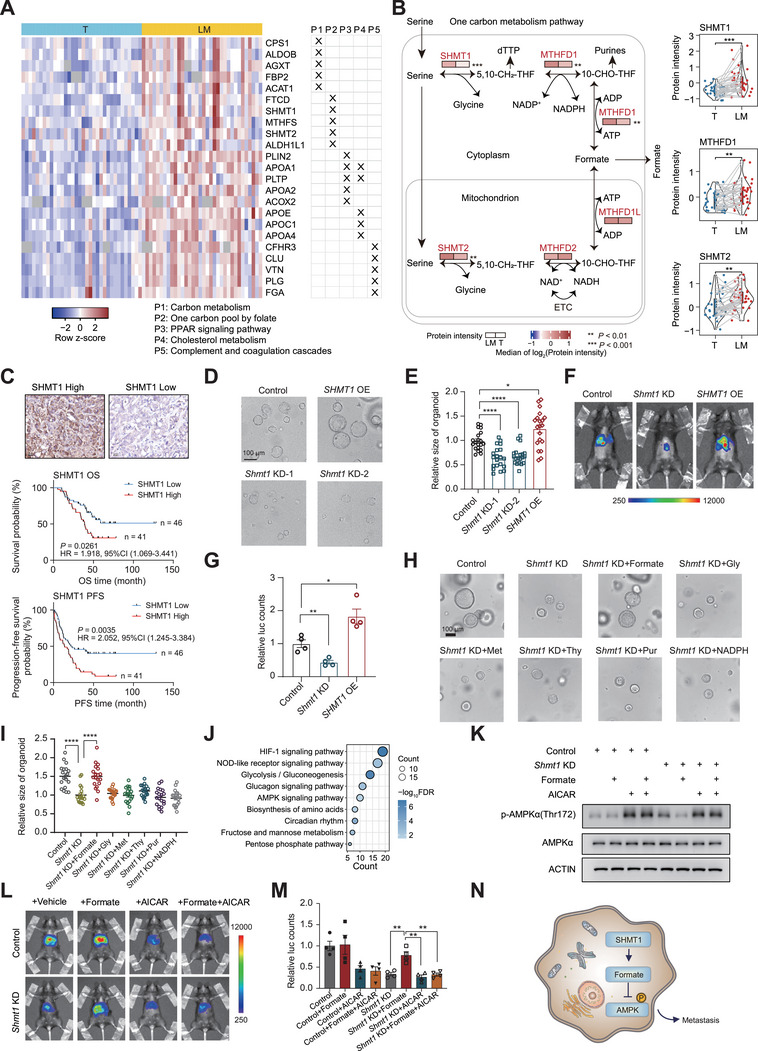
Proteomics revealed that SHMT1 regulated CRC tumorigenesis and metastasis. A) Top five changed proteins between LM and T samples in representative pathways of carbon metabolism, one carbon pool by folate, PPAR signaling pathway, cholesterol metabolism, and complement and coagulation cascades (two‐sided Wilcoxon Rank Sum and Signed Rank Tests, *p* < 0.05). B) The protein expressions of metabolic enzymes in one carbon pathway. The protein abundance was labeled in T and LM, respectively (two‐sided Wilcoxon rank sum and signed rank tests). C) Representative immunohistochemical staining for SHMT1 in the independent validation cohort of LM samples (*n* = 87) from FUSCC (Top). Scale bar, 20 µm. Kaplan‐Meier plot of OS and PFS stratified by SHMT1 protein level in the LM samples (log‐rank test, *n* = 87). D,E) Representative images and quantification of CRC organoid size with *Shmt1* knockdown and overexpression. Scale bar, 100 µm. F,G) Representative images and quantification of liver metastasis tumor in the intrasplenic injection mouse model. H,I) Representative images and quantification of *Shmt1* KD CRC organoid size supplemented with different metabolites. Scale bar, 100 µm. J) KEGG pathway enrichment analysis of genes that were significantly upregulated (FC > 1.2, *p* < 0.05) in *Shmt1* KD cells compared with both control and formate‐rescued *Shmt1* KD cells using transcriptomic data. K) Western blotting analyses of phosphorylated AMPKα level in CRC organoids with 1 mm AICAR or 1 mM formate for 24 h treatment. The protein expression of actin was used as the loading control. L,M) Representative images and quantification of liver metastasis tumor in the intrasplenic injection model. The control group and *Shmt1* KD group were treated with 125 mM formate in drinking water daily or 50 mg kg^−1^ AICAR i.p. every other day. N) A scheme showed SHMT1 promoted CRC tumorigenesis and metastasis via formate‐mediated AMPK inhibition. Data are presented as mean ± SEM. Two‐tailed Student's *t*‐test was performed. ^∗^ represented *p* < 0.05, ^∗∗^ represented *p* < 0.01, ^∗∗∗^ represented *p* < 0.001, ^∗∗∗∗^ represented *p* < 0.0001 and ns represented not significant.

Our proteomics analysis revealed that metabolism‐related pathways were highly enriched in liver metastasis samples (**Figure** **3**A), including carbon metabolism, one‐carbon pool by folate, PPAR signaling pathway, and cholesterol metabolism (FDR < 0.05). To validate these findings, we generated a mouse experimental model (Figure S3A, Supporting Information), and then collected CRC cells from the cecum (primary tumor) and liver metastases for proteomic analysis. KEGG enrichment analysis of differentially expressed proteins (fold change > 1.2, Wilcoxon rank sum and signed rank tests, *p* < 0.05) between liver metastases and primary tumors revealed a similar enrichment of carbon metabolism pathways in mouse liver metastases (Figure S3B, Supporting Information), consistent with findings in clinical samples (Figure 2A and 3A). Our proteomics data showed that three enzymes in one carbon metabolism, SHMT1, MTHFD1, and SHMT2, were significantly higher in LM samples than those in T samples (Figure 3B). Moreover, high expression of SHMT1 in LM samples was demonstrated to significantly associate with poor OS rate and progression‐free survival (PFS) rate in our independent validation cohort (Figure 3C, *n* = 87), and the detailed information was enclosed in Table S1 (Supporting Information).

To validate these findings, we generated intestinal KAP organoids from *Villin* ^CreERT2^, *Kras* ^LSL‐G12D^, *Apc* ^min/+^, *Trp53* ^flox/flox^ mice, following two months of tamoxifen (TAM) treatment. The results showed *Shmt1* knockdown (KD) impaired KAP organoid growth, while *SHMT1* overexpression yielded a contrasting outcome (Figure 3D,E; Figure S3C, Supporting Information). Whole‐mount staining of KAP organoids revealed that *Shmt1* KD led to reduced proliferation signals (pH3⁺) and increased apoptosis signals (CC3⁺) compared to the control group. In contrast, *SHMT1* overexpression resulted in a higher pH3⁺ cell ratio (Figure S3F–H, Supporting Information). These isogenic, luciferase‐labeled organoids were implanted into immunocompetent C57BL/6 wild‐type (WT) mice via the intrasplenic route (i.s.). We demonstrated that *Shmt1* KD attenuated metastatic progression, as evidenced by bioluminescence imaging quantification and histological evaluation (Figure 3F,G; Figure S3K,L, Supporting Information). Similarly, the role of CPS1 was validated using the same approach (Figure S3C–J, Supporting Information). To identify key downstream metabolites regulated by SHMT1 in CRC progression, we conducted metabolite re‐supplementation experiments in *Shmt1* KD organoids. Unexpectedly, only exogenous formate largely reversed the inhibitory effects observed in *Shmt1* KD organoids (Figure 3H,I). We further verified that the formate level in mouse liver metastasis was lower in *Shmt*1 KD group and higher in *SHMT*1 overexpression group compared to that in the WT group (Figure S3M, Supporting Information). Based on this, we hypothesized that SHMT1‐catalyzed formate might regulate downstream effectors involved in metastasis. To explore the pathways influenced by formate, we conducted transcriptomic analysis of *Shmt1* KD cells with or without formate supplementation (Figure S3N, Supporting Information). KEGG pathway enrichment analysis further revealed that formate supplementation significantly inhibited the HIF‐1 signaling, AMPK signaling, and pentose phosphate pathways (Figure 3J), while upregulating the Wnt and Hippo signaling pathways (Figure S3O, Supporting Information). Western blot validation confirmed that *Shmt1* KD organoids exhibited elevated phosphorylated AMPK level, which was reduced by formate but further increased by the AMPK agonist AICAR (Figure 3K). To investigate this effect in vivo, we administered either 125 mM formate in drinking water or intraperitoneal AICAR injections (50 mg kg^−1^) to tumor‐bearing mice with *Shmt1* KD or control tumors. As expected, liver tumor burden increased in the formate supplementation group, and this effect was mitigated by AICAR treatment (Figure 3L,M). Collectively, these findings suggest that SHMT1 promotes CRLM by inhibiting AMPK signaling through formate‐mediated mechanisms (Figure 3N).

### 2.5 Phosphoproteomics Identifies the Role of NDRG1 Ser330 Phosphorylation‐Dependent Degradation in Liver Metastasis

**Figure 4:**
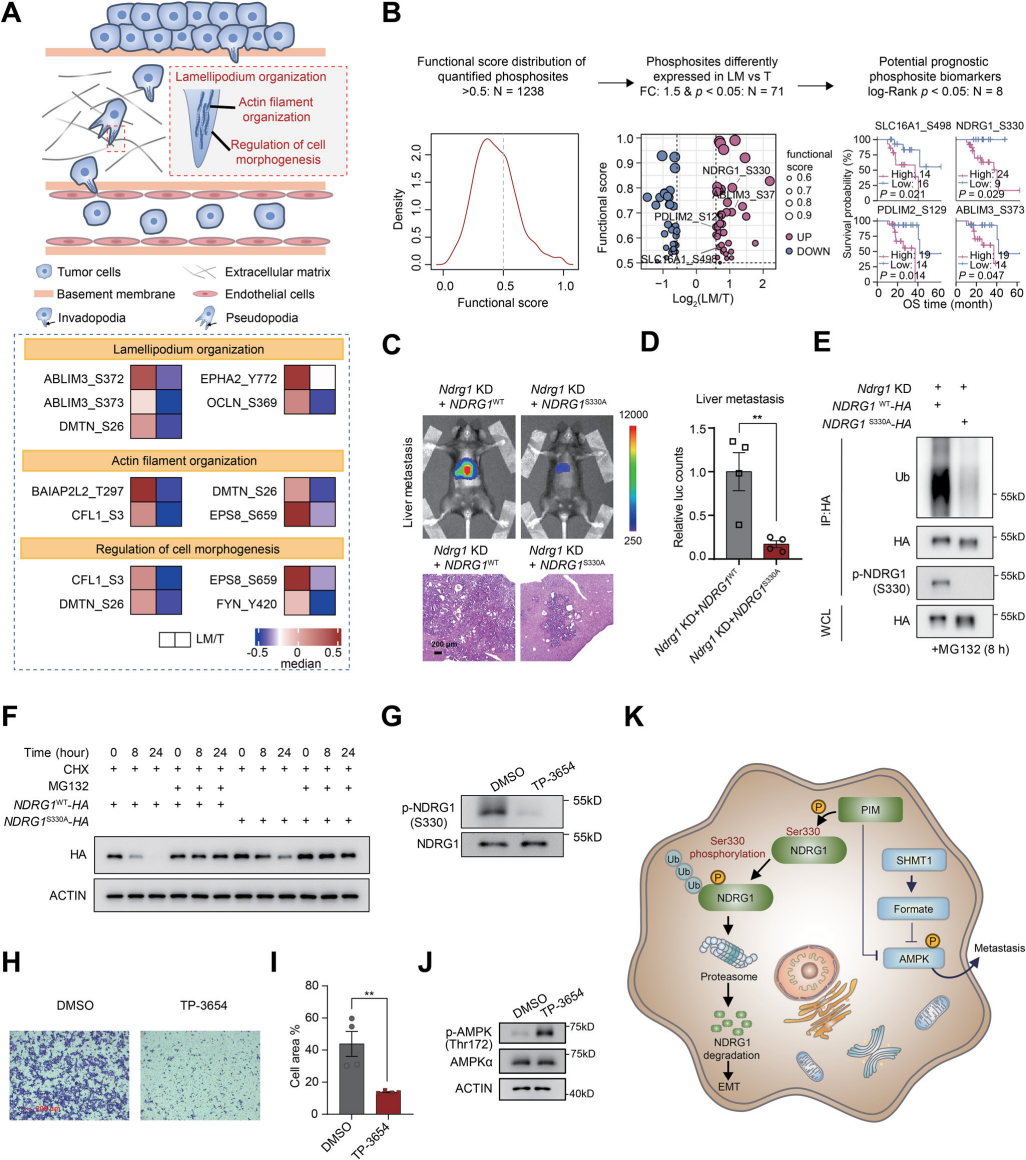
Phosphoproteomics revealed the role of phosphorylated NDRG1 on Ser330 in liver metastasis. A) Schematic illustration of actin filament organization, regulation of cell morphogenesis, and lamellipodium organization (upper panel) with related phosphosite abundance (bottom). B) The criteria to identify functional phosphosites related to liver metastasis. C,D) Representative images and quantification of liver metastasis tumor with *NDRG1* S330A mutant and WT in the intrasplenic injection model. E) *NDRG1* S330A mutant decreased the ubiquitination level of NDRG1. IB analysis of WCLs and anti‐HA IP of the indicated cells with or without *NDRG1* S330A mutant. F) *NDRG1* S330A mutant increased the stability of NDRG1. Control or *NDRG1* S330A mutant cells were treated with 50 µg mL^−1^ cycloheximide (CHX) and 20 µm MG132 for 0, 8, and 24 h. Protein extracts were immunoblotted for the indicated proteins. G) Western blotting analyses of phosphorylated NDRG1 level (normalized to NDRG1 protein expression level). KAP cells were treated with 3 µM of pan PIM inhibitor TP‐3654 for 1 h. H,I) Image and quantification of migrated cells in the transwell assay with or without 3 µM TP‐3654 treatment for 18 h. J) Western blotting analyses of phosphorylated AMPKα level upon 3 µM TP‐3654 treatment for 1 h. The protein expression of actin and AMPKα was used as the loading control. K) Proposed model for PIM‐dependent Ser330 phosphorylation of NDRG1. Increased level of phosphorylated NDRG1 on Ser330 facilitated further ubiquitination and degradation in liver metastasis. Data are presented as mean ± SEM. Two‐tailed Student's *t*‐test was performed. ^∗^ represented *p* < 0.05, ^∗∗^ represented *p* < 0.01, ^∗∗∗^ represented *p* < 0.001 and ns represented not significant.

Our phosphoproteomics showed that LM samples significantly affected phosphorylation events in actin filament organization, regulation of cell morphogenesis, and lamellipodium organization with a variety of significantly changed phosphorylation sites (**Figure** 2B and **4**A), which were featured in a specific motif (Figure S4A, Supporting Information). We then filtered crucial CRLM phosphosites and identified 5 upregulated and 3 downregulated phosphosites with high functional score^[^ ^41^ ^]^ and prognostic relevance (log‐rank *p* value < 0.05) (Figure 4B, Table S5, Supporting Information). Among them, Ser498 phosphorylation on monocarboxylate transporter 1 (SLC16A1), Ser330 phosphorylation on NDRG1, and Ser129 phosphorylation on PDZ and LIM domain protein 2 (PDLIM2) were highly conserved among different species (Figure S4B, Supporting Information), suggesting their potentially functional roles.

Recent studies demonstrated NDRG1 was a potent growth and metastasis suppressor in multiple cancers.^[^ ^42^, ^43^ ^]^ In our LM samples, the phosphorylation level of NDRG1 Ser330 (NDRG1 S330p) was positively correlated with actin cytoskeleton reorganization (ρ = 0.43, *p* = 0.013, Spearman correlation analysis) and actin filament‐based movement (ρ = 0.34, *p* = 0.053) (Figure S4C, Supporting Information). To elucidate the impact of NDRG1 Ser330 phosphorylation on liver metastasis in CRC, we introduced a mutation at Ser330 to alanine in KAP cells to abrogate its phosphorylation. Consistent with our hypothesis, mice injected with KAP cells harboring the *NDRG1* Ser330 mutant exhibited significantly reduced burden of liver metastasis in intrasplenica injection model (Figure 4C,D). Between the high and low CRS patient groups, no significant differences in NDRG1 Ser330 phosphorylation were observed in either T or LM samples (Figure S4D, Supporting Information). NDRG1 protein expression showed a negative correlation with the EMT pathway in our T and LM samples (ρ = −0.2, *p* = 0.098, Spearman correlation analysis), which was consistent with its role as a tumor suppressor^[^ ^44^, ^45^ ^]^ (Figure S4E, Supporting Information). Intriguingly, the protein level of NDRG1 correlated positively with the protein monoubiquitination process (ρ = 0.52, *p* = 0.002) and protein hydrolysis process based on SCF (Skp1/Cul1/F‐box protein) complex and proteasome (ρ = 0.39, *p* = 0.024) in LM samples (Figure S4F, Supporting Information). Additionally, GO BP enrichment analysis of NDRG1 interaction proteins retrieved from BioGRID database^[^ ^46^ ^]^ showed their enrichment in protein proteolysis, protein stability, and proteasomal protein catabolic process (Figure S4G, Supporting Information). Given the opposing roles of NDRG1 protein and its Ser330 phosphorylation in migration, we hypothesized that Ser330 phosphorylation was essential for NDRG1 ubiquitination and subsequent degradation. As expected, *NDRG1* ^WT^ cells exhibited significantly higher level of ubiquitination than *NDRG1* ^S330A^ mutant cells (Figure 4E). Cycloheximide (CHX) chase assay demonstrated that the *NDRG1* ^S330A^ mutant exhibited enhanced protein stability and a prolonged half‐life, indicating a critical role of Ser330 phosphorylation in regulating NDRG1 degradation. Moreover, treatment with the proteasome inhibitor MG132 resulted in NDRG1 accumulation, confirming that its degradation depends on the ubiquitin‐proteasome pathway (Figure 4F).

To identify the kinase responsible for NDRG1 Ser330 phosphorylation, we utilized a publicly available resource^[^ ^47^ ^]^ to predict the potential kinase‐substrate relationship. As a result, the PIM family (PIM1, PIM2, PIM3) showed the highest possibility to phosphorylate NDRG1 at Ser330 (Figure S4H, Supporting Information). Consistently, our transcriptomic analysis revealed significantly higher PIM1 expression in LM samples compared to T samples (Figure S4I, Supporting Information). Further, we treated CRC cells with the pan‐PIM inhibitor TP‐3654 to assess whether PIM kinase activity affects NDRG1 phosphorylation at Ser330. Notably, treatment with 3 µM TP‐3654 led to a significant reduction in NDRG1 phosphorylation at S330 (Figure 4G; Figure S4J, Supporting Information). Additionally, TP‐3654 treatment suppressed the migration and invasion abilities of CRC cells (Figure 4H,I). Given that PIM kinases are known to negatively regulate AMPK activation,^[^ ^48^ ^]^ we observed increased AMPK phosphorylation in TP‐3654‐treated CRC organoids, further suggesting that PIM negatively regulates AMPK activation (Figure 4J). Together, our results indicate that PIM‐dependent NDRG1 Ser330 phosphorylation promotes NDRG1 ubiquitination and degradation, thereby facilitating liver metastasis in CRC (Figure 4K).

### 2.6 Proteomics Identifies CRLM Patient Subtypes

**Figure 5:**
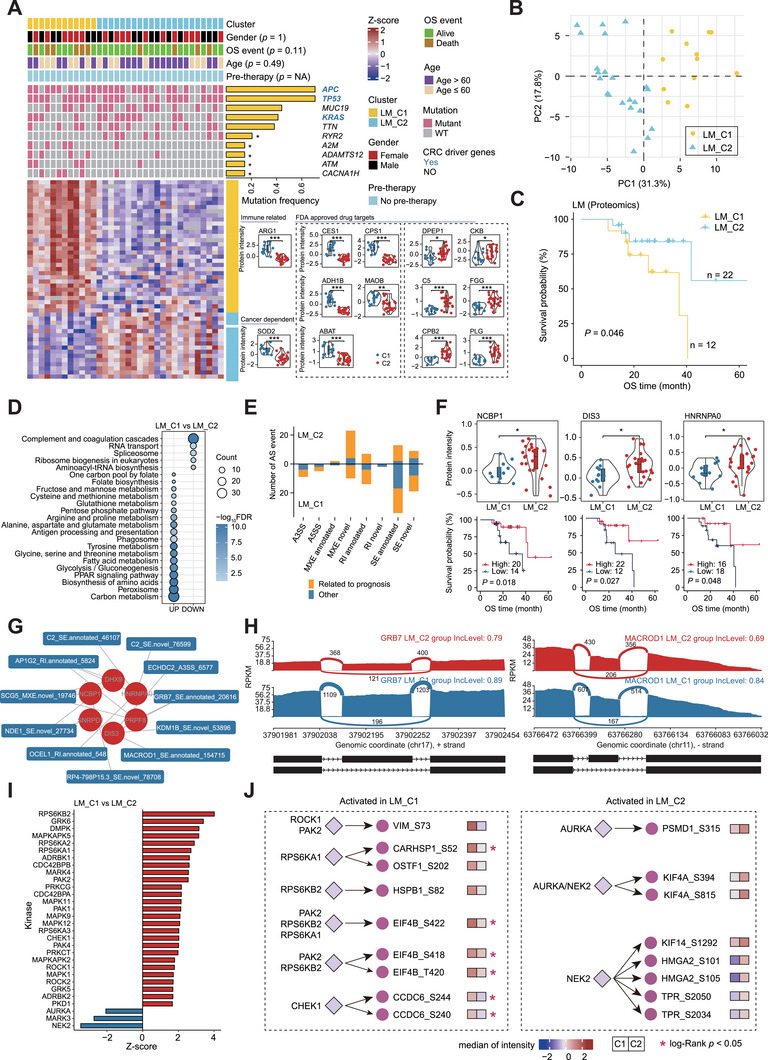
Proteomic subtypes of CRLM patients. A) Heatmap showing the top 5 high‐frequency mutated genes and low‐frequency mutated genes with significant difference (mutation frequency ≤ 15%), and significantly different expressed proteins. For comparison of gene mutation frequency and protein expression between C1 and C2 subtypes, Fisher's exact test and two‐sided Wilcoxon rank sum and signed rank test were performed, respectively. B) Principal component analysis (PCA) for the most variant proteins (MAD > 1). C) C1 subtype correlated with poor prognosis. Kaplan‐Meier curve of OS in patients with C1 and C2 subtypes (log‐rank test, *n* = 34). D) Representative KEGG pathways enriched in C1 and C2 subtypes based on proteomic data. E) The number of significantly changed AS events in C1 and C2 subtype samples (IncLevelDifference > 0.1, FDR < 0.05) associated with or without good/poor OS (Kaplan‐Meier analysis, log‐rank test, *p* < 0.05). F) Boxplot and violin plot showed the protein intensity of splicing‐related factors in C1 and C2. Kaplan‐Meier curves of OS in patients with high and low NCBP1/DIS3/HNRNPA0 protein expression (two‐sided Wilcoxon rank sum and signed rank tests, *p* < 0.05, log‐rank test, *n* = 34). G) The correlation network (Spearman correlation, FDR < 0.05) between splicing‐related factors and significantly different AS events. H) Sashimi plot of representative AS events between C1 and C2 subtypes. I,J) Kinase activities in C1 (red) and C2 (blue) subtype samples were evaluated by KSEA (*p* < 0.05), and representative kinase‐substrate relationships were annotated. ^∗^ represented *p* < 0.05, ^∗∗^ represented *p* < 0.01, ^∗∗∗^ represented *p* < 0.001 and ns represented not significant.

Although mCRC presented a poor clinical outcome, prognostic difference was evidently observed among the CRLM.^[^ ^6^, ^8^, ^10^, ^49^ ^]^ We then performed unsupervised clustering using the proteomic data of LM and T samples, respectively. Compared with T samples, two clusters (C1 and C2) were clearly identified in LM samples (**Figure** **5**A; Figure S5A,B, Supporting Information). Unsupervised clustering based on phosphoproteomics or transcriptomics data showed less unambiguous in LM samples than based on the proteomics data (Figure S5C, Supporting Information). In addition, the PCA result displayed a clear separation between C1 and C2 subtypes (Figure 5B). Of particular note, the two proteomic subtypes showed a significant difference (log‐rank *p* value < 0.05) in OS rate (Figure 5C; Figure S5A, Supporting Information). Conversely, no significant differences in OS rate between phosphoproteomics‐ or transcriptomics‐based subtypes were observed (Figure S5D, Supporting Information).

To further characterize the molecular difference between the two subtypes, we performed a comprehensive multi‐omics comparison. There were no significant differences of the high‐frequence mutated genes (mutation frequency > 30%) between LM C1 and C2 subtypes, including the CRC driver genes *APC*, *TP53*, and *KRAS* (Figure 5A), while 17 low‐frequence mutated genes (mutation frequency ≤ 15%) showed significant difference (fisher exact test, *p* < 0.05), indicating small genomic difference between C1 and C2 subtypes. In contrast, at the proteomic level, there were obviously distinct protein signatures and signaling pathways. KEGG enrichment analysis showed the C1 subtype was closely related to a variety of metabolism pathways (FDR < 0.05), such as carbon metabolism, fatty acid metabolism, and amino acid metabolism, whereas the C2 subtype was enriched with RNA‐related functions, such as RNA transport and splicing (Figure 5D). To gain deeper insights into the RNA splicing pattern of these two subtypes, we performed the transcriptomics‐based AS analysis. In total, we identified 226107 AS events in LM samples, including 10934 A3SS, 7085 A5SS, 37125 MEX, 5393 RI, and 165570 SE. Among them, 148 AS events showed a significant difference between C1 and C2 subtypes (FDR < 0.05 and absolute IncLevelDifference > 0.1), of which *KHK*, *RPP21*, and *U2AF1L4* were the top three genes with the highest number of significantly different AS events (Figure 5E; Figure S5E, Supporting Information). Survival analysis pinpointed that 98 out of 148 splicing events were correlated with either good or poor prognosis (Figure 5E). AS is processed in the spliceosome and regulated by a panel of RNA splicing regulators. To conduct a more in‐depth analysis of the AS regulatory network, we next utilized our proteomics data to explore the AS regulatory factors according to a recent publication by Rogalska et al.^[^ ^50^ ^]^ between C1 and C2 subtypes. The results showed the protein expression levels of 17 splicing‐related factors were significantly upregulated in the C2 subtype, whereas 2 were upregulated in the C1 subtype (fold change > 1.2 and *p* < 0.05, Table S6, Supporting Information). Among them, high protein levels of DHX9, DIS3, HNRNPA0, NCBP1, PRPF8, and SNRPD1 were associated with good prognosis (log‐rank *p* < 0.05), which was consistent with C2 subtype‐enriched splicing pathways and good clinical outcome (Figure 5F; Figure S5F, Supporting Information). Notably, *NCBP1*, *PRPF8*, and *SNRPD1* were dependent genes in both CRC and hepatocellular carcinoma. Furthermore, Spearman correlation analysis suggested the regulatory network (positive and negative) between 6 splicing factors and 11 AS events (FDR < 0.05) (Figure 5G,H; Figure S5G, Supporting Information). Among them, DIS3, HNRNPA0, and PRPF8 were negatively correlated (FDR < 0.05) with the AS event of growth factor receptor‐bound protein 7 (GRB7), a gene reported to be overexpressed in invasive and metastatic cancer tissues.^[^ ^51^ ^]^ Given that mRNA splicing can generate variants with different functions or shift the balance among isoforms, further biological investigation of the role of mRNA splicing isoforms in CRC metastasis is required.

Phosphoproteomics inferred C2 subtype enriched more biological processes, such as cell junction organization, chromatin remodeling, actin filament‐based movement, and post‐translational protein modification in LM samples (Figure S5H, Supporting Information). KSEA revealed multiple kinases, including RPS6KA1, RPS6KB2, PAK1, PAK2, PAK4, CHEK1, ROCK1 and MAPK1 showed higher activities in C1 than those in C2 samples (Figure 5I). Among these hyperactivated kinases in C1, RPS6KA1, RPS6KB2, and PAK2 were predicted to regulate the Ser422 phosphorylation of eukaryotic translation initiation factor 4B (EIF4B) (Figure 5J). EIF4B, which plays a vital role in the translational initiation by recruiting the ribosome to mRNA, can be phosphorylated at Ser422 by RPS6KA1 and RPS6KB2.^[^ ^52^, ^53^ ^]^ These discrepancies of abnormally activated kinases between C1 and C2 subtypes may contribute to distinct molecular signatures, signaling pathways, and clinical outcomes. The immune scores and stromal scores between C1 and C2 were not statistically different at both the transcriptomic and protein levels (Figure S5I, Supporting Information). Nevertheless, ARG1 was strikingly increased in the C1 subtype (Figure 5A; Figure S5J, Supporting Information) and showed the potential to correlate with poor prognosis in our T and LM samples (Figure S2I, Supporting Information).

### 2.7 Subtype‐Specific Biomarker Identification

**Figure 6:**
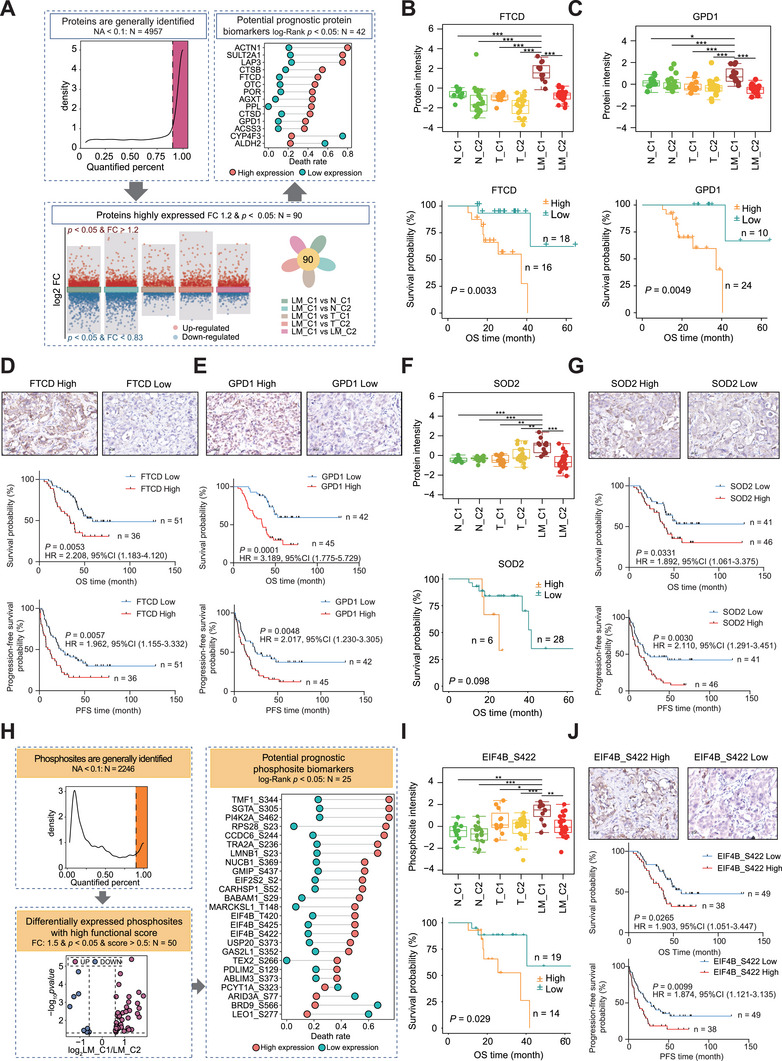
Identification and validation of potential prognostic biomarkers. A) The workflow to identify potential subtype‐specific protein biomarkers. B–G) FTCD (B), GPD1 (C), and SOD2 (F) were highly expressed in liver metastasis of C1 subtype (two‐sided Wilcoxon rank sum and signed rank test) with clinical prognostic relevance (log‐rank test, *n* = 34). Representative images of immunohistochemical staining for FTCD (D), GPD1 (E), and SOD2 (G) in the independent validation cohort (*n* = 87). Scale bar, 20 µm. Kaplan‐Meier plot of OS and PFS stratified by protein level in the liver metastasis samples (*n* = 87). H) The workflow to identify subtype‐specific prognostic phosphosites. I) The high level of EIF4B Ser422 (EIF4B_S422) phosphorylation in liver metastasis of C1 subtype (two‐sided Wilcoxon rank sum and signed rank test) with clinical prognosis relevance (log‐rank test, *n* = 33). J) Representative images of immunohistochemical staining for EIF4B_S422 phosphorylation in the independent validation cohort (*n* = 87). Scale bar, 20 µm. Kaplan‐Meier plot of OS and PFS stratified by EIF4B_S422 phosphorylation in LM samples (log‐rank test, *n* = 87). ^∗^ represented *p* < 0.05, ^∗∗^ represented *p* < 0.01, ^∗∗∗^ represented *p* < 0.001 and ns represented not significant.

To uncover subtype‐specific prognostic proteins (**Figure** **6**A), we required the protein candidates that were generally expressed with a missing value < 10% across our samples. We then calculated the differentially expressed proteins between the proteins in the C1 subtype of LM and those in all the other sample sets (i.e., the C2 subtype of LM, the C1 subtype of T, the C2 subtype of T, the C1 subtype of N, or the C2 subtype of N), respectively (Wilcoxon rank sum and signed rank tests). Subsequently, 90 higher expression proteins in the C1 subtype of LM samples (fold change > 1.2 and *p* < 0.05) than all the other sample sets were considered for further analysis (Figure S6A, Supporting Information). Among them, the one‐carbon metabolic process was the top‐enriched pathway using the KEGG database (Figure S6A, Supporting Information). Forty‐two proteins of them were significantly correlated with prognosis (log‐rank *p* < 0.05) and considered as potential prognostic biomarkers (Figure 6A; Figure S6B and Table S7, Supporting Information). Considering the prognostic relevance (*p* < 0.01), subcellular location, protein classification (functional enzyme), and antibody availability for immunohistochemical staining (Figure S6C,D, Supporting Information), we next chose formimidoyltransferase‐cyclodeaminase (FTCD) and glycerol‐3‐phosphate dehydrogenase 1 (GPD1) for further investigation. Our proteomics data showed that the protein expressions of FTCD and GPD1 were higher in C1 subtype LM samples than in all the other sample sets, and correlated with clinical prognosis (Figure 6B,C). In the independent validation LM cohort (*n* = 87), high expression of FTCD and GPD1 was also significantly (OS, log‐rank *p* < 0.05, PFS, log‐rank *p* < 0.05) correlated with poor prognosis (Figure 6D,E). To further explore subtype‐enriched functional proteins, we retrieved the hepatocellular carcinoma or CRC‐dependent genes from the DepMap database (Table S8, Supporting Information), yielding 1310 and 1298 genes, respectively. Among these genes, we extracted proteins highly expressed in the C1 subtype of LM samples compared with their paired T samples (see Supplementary Methods). The results showed that superoxide dismutase 2 (SOD2) was the most significantly upregulated protein in the C1 subtype of LM samples (Figure S6E, Supporting Information). Interestingly, SOD2 was only highly expressed at the protein level, but not at mRNA level (Figure 6F; Figure S6F, Supporting Information). Meanwhile, high expression of SOD2 in LM samples correlated with poor prognosis (OS, log‐rank *p* < 0.05; PFS, log‐rank *p* < 0.05) in the independent validation cohort (*n* = 87) (Figure 6G).

To discover prognostic phosphosites, we similarly required the phosphosite candidates that were generally expressed with a missing value < 10% and differentially changed between C1 and C2 subtypes. In total, we identified 108 upregulated and 60 downregulated phosphosites in C1 (fold change > 1.5, Wilcoxon rank sum and signed rank tests, *p* < 0.05). After integrating potential regulatory functional score (functional score > 0.5)^[^ ^41^ ^]^ and prognostic information, we finally identified 25 phosphosites with statistically significant prognostic relevance between the two subtypes (log‐rank *p* < 0.05, Figure 6H; Table S7, Supporting Information). Among them, EIF4B_S422 and EIF4B_T420 phosphorylation were not only specifically highly expressed in the C1 subtype of LM samples (*p* < 0.05), but also significantly correlated with poor prognosis (log‐rank *p* < 0.05, Figure 6I; Figure S6G, Supporting Information). In contrast, this result was not observed at mRNA and protein levels (Figure S6H, Supporting Information). Ser422 phosphorylation on EIF4B was reported to be regulated by mTOR/PI3K and MAPK pathways, and played physiological roles in the interaction of EIF4B with the eukaryotic translation initiation factor 3.^[^ ^53^, ^54^ ^]^ We next chose EIF4B_S422 phosphorylation for further validation in the independent cohort (*n* = 87). The results showed that a high level of EIF4B_S422 phosphorylation was significantly (OS, log‐rank *p* = 0.0265; PFS, log‐rank *p* = 0.0099) correlated with CRLM poor prognosis (Figure 6J).

## 3 Discussion

The genomics and proteomics studies of primary CRC samples have greatly advanced the understanding of molecular mechanisms, subtype classification, and precise therapies. Here, our proteogenomic study revealed that, in the liver metastasis sample, a large number of cellular pathways were dysregulated at the proteomic and phosphoproteomic levels but not the genomic level, notably those involved in the carbon metabolism pathway and actin cytoskeleton dynamics. Mechanistically, we further demonstrated that SHMT1 promoted CRC tumorigenesis and metastasis via formate‐mediated AMPK inhibition, and a role of PIM kinase‐regulated NDRG1 Ser330 phosphorylation induced degradation in CRLM. We further unveiled two distinct liver metastasis proteomic subtypes, C1 (metabolism) and C2 (RNA function), with distinct clinical outcomes. Finally, we identified several protein or phosphorylation prognostic biomarkers, including FTCD, GPD1, SOD2, and EIF4B S422 phosphorylation, which were validated in an independent CRLM patient cohort.

Metastatic cells often exploit reprogrammed metabolic pathways in their new microenvironment, particularly in metabolically active organs like the liver.^[^ ^15^, ^38^, ^39^ ^]^ This adaptation supports their survival, growth, and colonization at secondary sites. In particular, the formate‐mediated one‐carbon metabolism pathway is necessary for the *de novo* synthesis of purines, thymidylate, and S‐adenosylmethionine (SAM). Cytoplasmic SHMT1 directs the allocation of folate‐activated one‐carbon units toward thymidylate or SAM biosynthesis.^[^ ^55^ ^]^ Since SAM is the major cellular methyl donor, dysfunction in this SHMT1‐driven pathway impairs cellular methylation and genome stability, thereby providing a mechanistic basis for elucidating the distinct contributions of these metabolic pathways to CRC risk.^[^ ^55^ ^]^ We demonstrated that SHMT1 played a crucial role in CRLM by regulating formate production, thereby modulating cellular energy metabolism, as evidenced by inhibited AMPK activity. Despite evidence that formate induces a metabolic switch that elevates adenine nucleotide level to promote glycolysis and repress AMPK activity,^[^ ^56^ ^]^ we acknowledge that the precise mechanism between formate and AMPK signaling remains to be defined.

The quantitative phosphoproteomics results indicate the significantly upregulated phosphosites in liver metastasis (Table S5, Supporting Information). These phosphosites may be involved in the processes of CRC cell migration and adaptation to the liver environment. Recent studies demonstrated that NDRG1 was a potent growth and metastasis suppressor in multiple cancers. It exhibited inhibitory effects on various cellular signaling pathways, such as the oncogenic RAS, PI3K/AKT, and WNT pathways, highlighting its potential as a new therapeutic target in cancer treatment.^[^ ^42^ ^]^ We uncovered a novel mechanism in which PIM kinase‐mediated phosphorylation of NDRG1 at Ser330 promoted its ubiquitination and subsequent degradation, thereby facilitating the liver metastasis of CRC. Besides, other studies demonstrated that the phosphorylation of Ser3 on CFL1 was a crucial event during EMT. CFL1 was responsible for modulating actin dynamics by promoting actin treadmilling, which also led to membrane protrusion, cell migration, and invasion. Phosphorylation of Ser3 on CFL1 reduced the expression of E‐cadherin and claudin‐3 in cell‐cell contacts while increasing the expression of vimentin protein in CRC cells.^[^ ^35^ ^]^ All this evidence suggested the reliability of our analysis. Nevertheless, the functional roles of the phosphorylation events remain to be further investigated.

Although PIM kinase‐mediated phosphorylation of NDRG1 at Ser330 and dephosphorylation of AMPK were verified by our experiments (Figure 4G and 4J), these regulations were not captured by KSEA (Figure 2C). The KSEA algorithm can only deduce the direct upstream kinase activity based on phosphoproteomics data. Indeed, PIM inhibited the phosphorylation of AMPKα at Thr172 (Figure 4J) in an indirect manner (i.e., AMPKα^T172^ is not the direct substrate of PIM).^[^ ^48^, ^57^, ^58^ ^]^ Therefore, the dephosphorylation of AMPK could not be detected by KSEA. The phosphorylation of NDRG1_S330 by PIM was not identified by KSEA because of the limited kinase‐substrate database, which did not include NDRG1_S330 as the substrate of PIM. Alternatively, we used another algorithm described by Sun et al.^[^ ^59^ ^]^ to calculate the kinase activity, which is able to employ a more comprehensive kinase‐substrate database (downloaded from PhosphoSitePlus database, version 6.7.5). The result showed that PIM1 activity was significantly elevated in liver metastasis samples (Figure not shown, Wilcoxon rank‐sum and signed‐rank tests, *p*‐value < 0.001). Together, these results further supported that PIM kinase activity was significantly up‐regulated in liver metastasis samples.

Unsupervised clustering of LM samples led to the identification of two subsets with distinct molecular characteristics and clinical outcomes, underscoring the molecular heterogeneity of CRLM and its potential for precise patient stratification and treatment. GPD1, which catalyzes the conversion of DHAP and NADH to G3P and NAD⁺, has been reported to be associated with poor prognosis in glioma.^[^ ^60^ ^]^ Additionally, high expression of SOD2 is associated with poor prognosis across multiple cancer types, including glioblastoma and oral squamous cell carcinoma.^[^ ^61^, ^62^ ^]^ In the CPTAC (Clinical Proteomic Tumor Analysis Consortium) datasets, higher expression of these molecules showed poor prognostic impact in glioblastoma multiforme (GBM for SOD2 and GPD1), lung adenocarcinoma (LUAD for GPD1), clear cell renal cell carcinoma (CCRCC for SOD2), and pancreatic ductal adenocarcinoma (PDAC for SOD2 and EIF4B_S422). These findings collectively support the reliability of these proteins or phosphosite as potential prognostic cancer biomarkers. Notably, although the relatively limited patient number (*n* = 34) in our multi‐omics dataset may lead to insufficient statistical power to identify some key molecules and pathways in CRLM, a larger independent cohort of LM samples (*n* = 87) was constructed for further validation. For instance, we observed that SOD2 exhibited relatively low statistical power (log‐rank *p* = 0.098) in discriminating clinical outcomes in our multi‐omics dataset (*n* = 34). Immunohistochemical staining in the validation cohort showed a significant correlation (log‐rank *p* = 0.0331) with poor prognosis.

In conclusion, our study offers a resource for CRLM study and provides new molecular insights and potential therapeutic targets for CRLM patients.

## 4. Experimental Section

### Clinical Sample Collection

The CRLM samples used for this study were collected from Fudan University Shanghai Cancer Center (FUSCC) from July 2012 to June 2019. Key eligibility criteria included patients pathologically diagnosed with colorectal liver metastases, without preoperative treatments, with synchronous or metachronous resection of the primary and liver metastases, and availability of matched primary tumor, liver metastasis, and adjacent normal colorectal tissues. Primary tumor tissues, matched liver metastasis tissues, and adjacent normal colorectal tissues (> 5 cm apart from the tumor edge) were surgically resected and transferred to sterile freezing vials. A total of 102 specimens were collected, along with complete clinical information, including gender, age, primary site, histological subtype, tumor node metastasis (TNM) staging, differentiation, tumor size, prognostic, and treatment information after surgery. The usage of clinical specimens was approved by the Ethical Committee and Institutional Review Board of FUSCC in compliance with ethical standards and patient confidentiality (approval numbers: 050432‐4‐1805C, Shanghai, China, 7 May 2018; 050432‐4‐1911D, Shanghai, China, 4 November 2019). Written informed consent was signed by all eligible patients.

### Statistical Analysis

GraphPad Prism 8.0 was used for statistical calculations. For all comparisons, a two‐tailed Student's *t* test (paired or unpaired) was performed. Data were presented as mean ± SEM. In all figures, ns represented not significant, ^∗^ represented *p* < 0.05, ^∗∗^ represented *p* < 0.01, and ^∗∗∗^ represented *p* < 0.001. For quantification of the signal density of positive cells in the tumor or organoid, images of tumor sections or organoids with IF or IHC staining were captured by a microscope. The positive cells were counted. Three fields in each were randomly selected for cell density analysis.

### Ethics Approval and Consent to Participate

This study involves human tissue samples, and the usage of clinical specimens was approved by the Ethical Committee and Institutional Review Board of FUSCC in compliance with ethical standards and patient confidentiality (approval numbers: 050432‐4‐1805C, Shanghai, China, 7 May 2018; 050432‐4‐1911D, Shanghai, China, 4 November 2019). Written informed consent was signed by all eligible patients. The usage of laboratory animals was approved by the Institutional biomedical research ethics committee of Shanghai Institute of Nutrition and Health, Chinese Academy of Sciences (approval numbers: SINH‐2021‐QJ‐2, Shanghai, China, 1 June 2021; SINH‐2022‐QJ‐2, Shanghai, China, 1 June 2022; SINH‐2023‐QJ‐2, Shanghai, China, 1 June 2023; SINH‐2024‐QJ‐2, Shanghai, China, 1 June 2024).

### Materials Availability

This study did not generate new unique reagents.

### Data and Code Availability

The datasets supporting the conclusions of this article are available in the iProx Consortium, [IPX0007391001], GSA database, [GSA database, https://ngdc.cncb.ac.cn/gsa/], and accession code PRJCA020890. The datasets supporting the conclusions of this article are included within the article and its Supplementary Tables.

This paper does not report original code.

Any additional information required to reanalyze the data reported in this work paper is available from the lead contact upon request.

## Conflict of Interest

The authors declare no conflict of interest.

## Author Contributions

W.Z., L.Z., Y.L., Z.L., Y.L., and X.W. contributed equally to this work. M.T., J.Q., and J.P. designed and supervised the study. W.Z., M.Z., and J.G. performed the omics experiments. Y.L. and X.W. performed the biological validation experiments. L.Z., Z.L., N.L., D.S., J.L., J.L., and F.W. contributed to omics data analyses. Y.L. and S.M. performed the TMA and pathology analyses. The manuscript was written by W.Z. and M.T., and revised by L.Z., J.N., S.L., B.L., J.P., and J.Q.

## Supporting information



Supporting Information

Supplemental Table 1

Supplemental Table 2

Supplemental Table 3

Supplemental Table 4

Supplemental Table 5

Supplemental Table 6

Supplemental Table 7

Supplemental Table 8

## Data Availability

The data that support the findings of this study are available in the supplementary material of this article.
