# Supplementary material for: Proteogenomic Characterization Reveals Metabolic Vulnerabilities and Aberrant Phosphorylation in Colorectal Metastasis to Liver
Source: Adv Sci (Weinh). 2025 Nov 6;13(4):e11744. doi: 10.1002/advs.202511744 (PMC12822478; doi:10.1002/advs.202511744)
Supplement: Supplementary file 1 — Supporting Information [file ADVS-13-e11744-s004.docx]

**Proteogenomic Characterization Reveals Metabolic Vulnerabilities and Aberrant Phosphorylation in Colorectal Metastasis to Liver**

Wensi Zhao^1,#^, Lei Zhao^1,#^, Yannan Lian^2,#^, Zhiwei Liu^3,#^, Yaqi Li^4,#^, Xuege Wang^2,#^, Mingya Zhang^3^, Ni Li^2^, Jingli Guo^3^, Danqing Shen^5^, Shaobo Mo^4^, Jiahao Li^6^, Linhui Zhai^1^, Jiahui Ni^3^, Sangkyu Lee^7^, Bin Liu^8^, Jing Li^5^, Fei Wang^6^, Junjie Peng^4,*^, Jun Qin^2,9*^, Minjia Tan^1,3,10,*^

^1^ Translational Research Institute of Brain and Brain-Like Intelligence, Shanghai Fourth People's Hospital, and Cancer Center, School of Medicine, Tongji University, Shanghai, 200434, China.

^2^ CAS Key Laboratory of Tissue Microenvironment and Tumor, Shanghai Institute of Nutrition and Health, Chinese Academy of Sciences, 320 Yueyang Road, Shanghai, 200031, China.

^3^ State Key Laboratory of Drug Research, Shanghai Institute of Materia Medica, Chinese Academy of Sciences, Shanghai, 201203, China.

^4^ Department of Colorectal Surgery, Fudan University Shanghai Cancer Center; Department of Oncology, Shanghai Medical College, Fudan University, Shanghai, 200032, China.

^5^ Department of Bioinformatics and Biostatistics, School of Life Sciences and Biotechnology, Shanghai Jiao Tong University, Shanghai, 200240, China.

^6^ Shanghai Key Laboratory of Intelligent Information Processing, School of Computer Science and Technology, Fudan University, Shanghai, 200433, China.

^7^ School of Pharmacy, Sungkyunkwan University, Suwon, 16419, Republic of Korea

^8^ Jiangsu Key Laboratory of Marine Pharmaceutical Compound Screening, College of Pharmacy, Jiangsu Ocean University, Lianyungang, 222005, China.

^9^ Jinfeng Laboratory, Chongqing, 401329, China.

^10^ Zhongshan Institute for Drug Discovery, Shanghai Institute of Materia Medica, Chinese Academy of Sciences, Zhongshan, 528400, China.

* Corresponding author: Junjie Peng (pengjj@shca.org.cn), Jun Qin (qinjun@sibs.ac.cn), Minjia Tan (mjtan@simm.ac.cn);

# These authors contributed equally.

**Supplementary Figures**

**
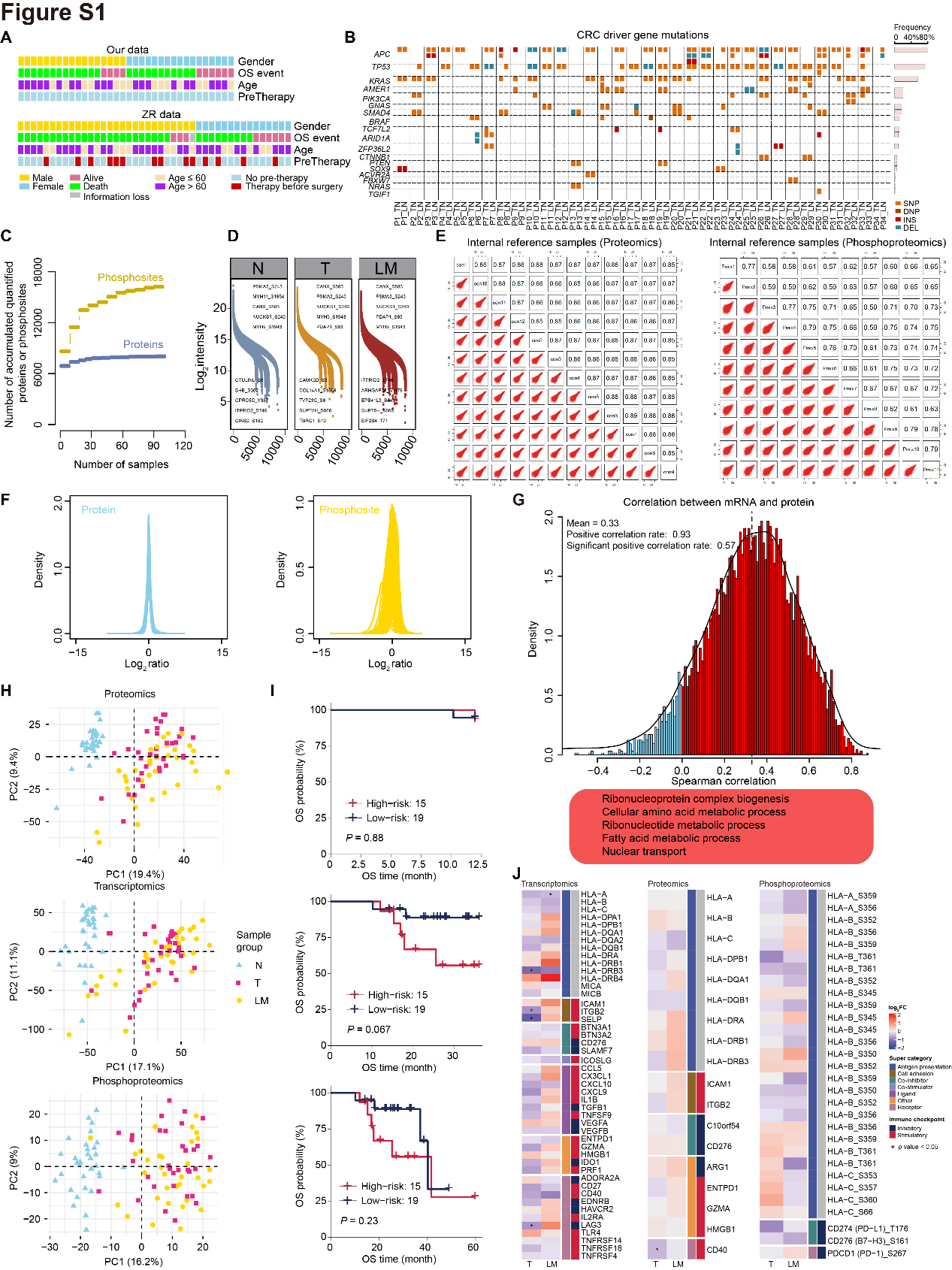
**

**Fig.S1.** Multi-omic landscape of CRLM. (A) Clinical information compared with a published study^1^. (B) Mutation information of CRC driver genes in T and LM samples. (C) Number of cumulative quantified proteins and phosphosites. (D) Distribution of phosphosite intensity with median normalization by corresponding proteomic sample and log2 transformation. The top five highest and lowest abundant phosphosites were listed in N, T and LM samples, respectively. (E) Pearson correlation coefficient between internal reference sample (IRS) at the proteome and phosphoproteome level. (F) Density distribution of protein or phosphosite abundance across all samples. (G) Distribution of gene-wise correlation between transcriptomics and proteomics data (Spearman correlation). GO BP enrichment analysis of positively correlated genes (FDR < 0.05 and ρ > 0.3). (H) PCA was performed at transcriptomic, proteomic and phosphoproteomic level, respectively. (I) Kaplan-Meier survival curves for 1-, 3-, and 5-year OS in patients with high versus low CRS (log-rank test, n = 34). (J) Heat map presenting mRNA, protein and phosphorylation site expression of immunomodulatory genes for patients with high versus low CRS (two-sided Wilcoxon rank sum and signed rank tests). ∗ represented P < 0.05.

**
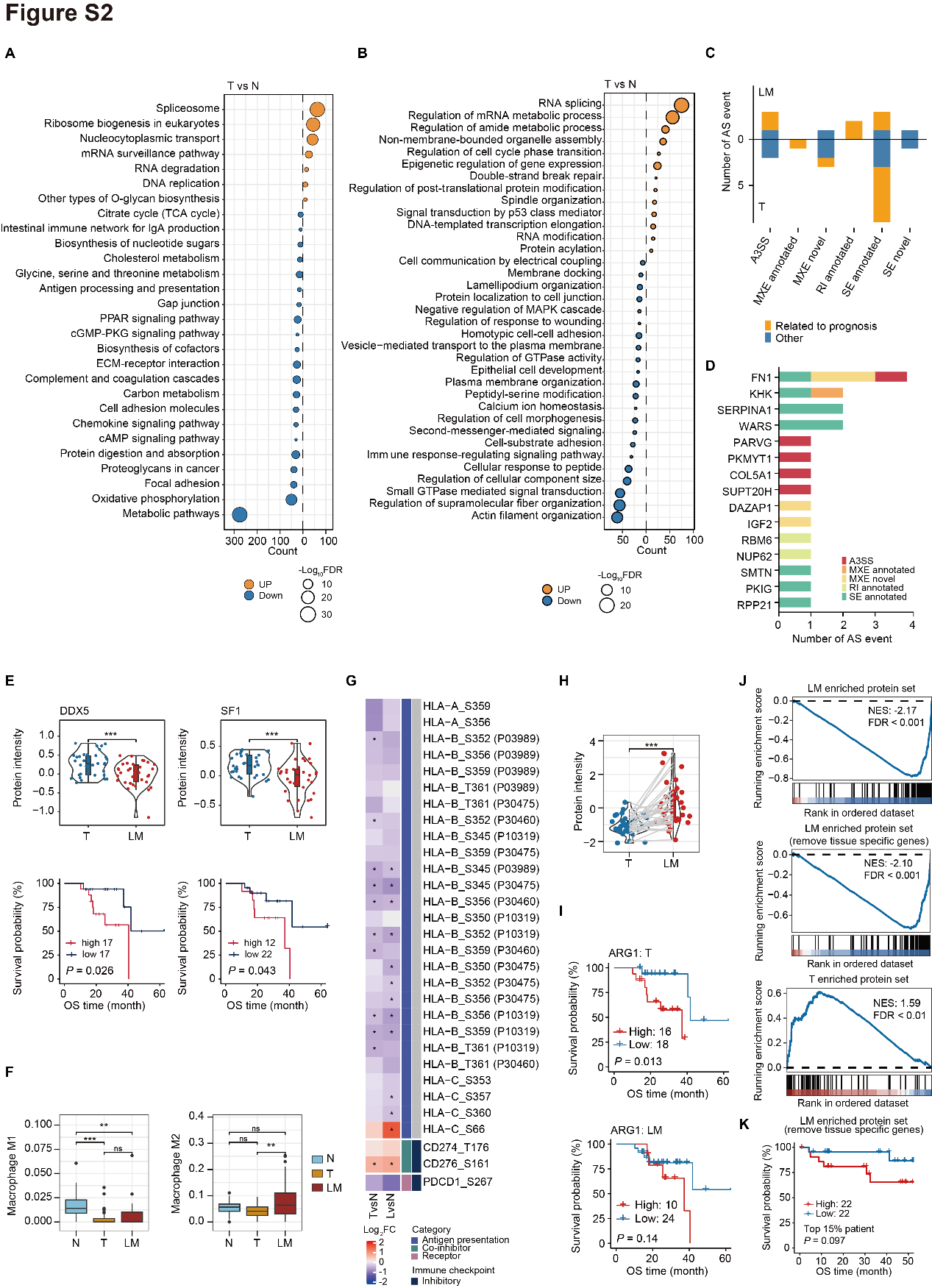
**

**Fig.S2.** Characterization of splicing events, immune signatures and potential risk gene sets. (A-B) KEGG and GO BP enrichment analysis were performed using proteomics and phosphoproteomics data between T and N samples, respectively (FDR < 0.05). (C) The number of significantly changed AS events between LM and T samples (absolute IncLevelDifference > 0.1, FDR < 0.05) associated with or without good/poor OS (Kaplan-Meier analysis, log-rank test, P < 0.05). (D) Genes with the top AS events that were significantly different between LM and T samples. (E) Boxplot and violin plot showed the protein intensity of splicing-related factors in T and LM samples (two-sided Wilcoxon rank sum and signed rank tests). Kaplan-Meier curves of OS in patients with high and low DDX5/SF1 protein expression (log-rank test, n = 34). (F) The relative abundances of macrophage M1 and M2 calculated using xCell at transcriptomic level (two-sided Wilcoxon rank sum and signed rank tests). (G) Heat map presenting the expression of phosphorylation sites of immunomodulatory genes (two-sided Wilcoxon rank sum and signed rank tests). (H) Violin plot based on the protein expression of ARG1 between T and LM samples (two-sided Wilcoxon rank sum and signed rank tests). (I) Kaplan-Meier plot of OS stratified by ARG1 protein level in T and LM samples (n = 34). (J) Gene set enrichment analysis using a publicly available proteomic data (Li et al. dataset^2^). LM enriched protein set (after more filter criteria) with and without tissue specific genes, and T enriched protein set (after more filter criteria) were utilized as the molecular signature database. (K) Kaplan-Meier curve of OS in patient from Li et al. dataset (log-rank test) using the median protein expression of LM enriched protein set (after more filter criteria) without tissue specific genes for each patient. ∗ represented P < 0.05, ∗∗ represented P < 0.01, ∗∗∗ represented P < 0.001 and ns represented not significant.

**
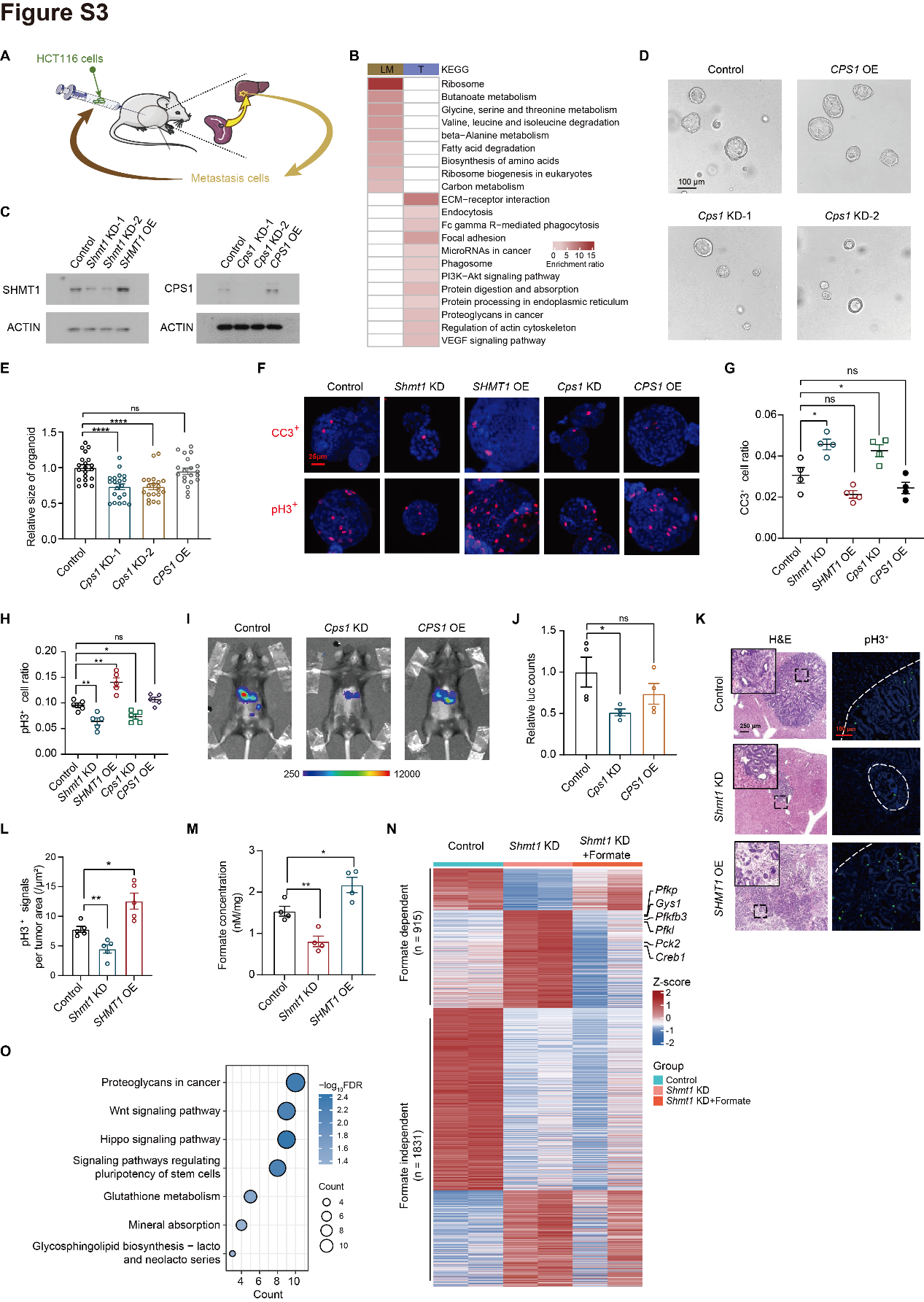
**

**Fig.S3.** Proteomics indicated the underlying mechanisms of CRC tumorigenesis and metastasis. (A) Mouse model construction for CRLM. (B) Representative KEGG pathways enriched in primary tumor and liver metastasis based on proteomic data in the mouse model (FDR < 0.05). (C) Western blotting analyses of SHMT1 and CPS1 expression. The protein expression of actin was used as the loading control. (D-E) Representative images and quantification of organoid size with *Cps1* knockdown and overexpression. Scale bar, 100 μm. (F-H) Immunofluorescence staining and quantification of cleaved-caspase3 positive (CC3^+^) and phospho-histone H3 positive (pH3^+^) signal cells. Scale bar, 25 μm. (I-J) Representative images and quantification of liver metastasis tumor in an intrasplenic injection model. (K) H&E-stained sections in the liver metastasis sections as indicated. Scale bars, 250 μm. (L) Immunofluorescence quantification of the liver metastasis with pH3^+^ signals. (M) Formate level measured by formate assay kit in liver metastasis as indicated. (N) Heatmap of gene expressions in KAP organoid with or without formate re-supplement. (O) KEGG pathway enrichment analysis (FDR < 0.05) of genes that were significantly downregulated (FC < 0.83, P < 0.05) in *Shmt1* KD cells compared with both control and formate-rescued *Shmt1* KD cells using transcriptomic data. For all comparisons, data are presented as mean ± SEM. Two-sided Student's t test was performed. ∗ represented P < 0.05, ∗∗ represented P < 0.01, ∗∗∗ represented P < 0.001 and ns represented not significant.

**
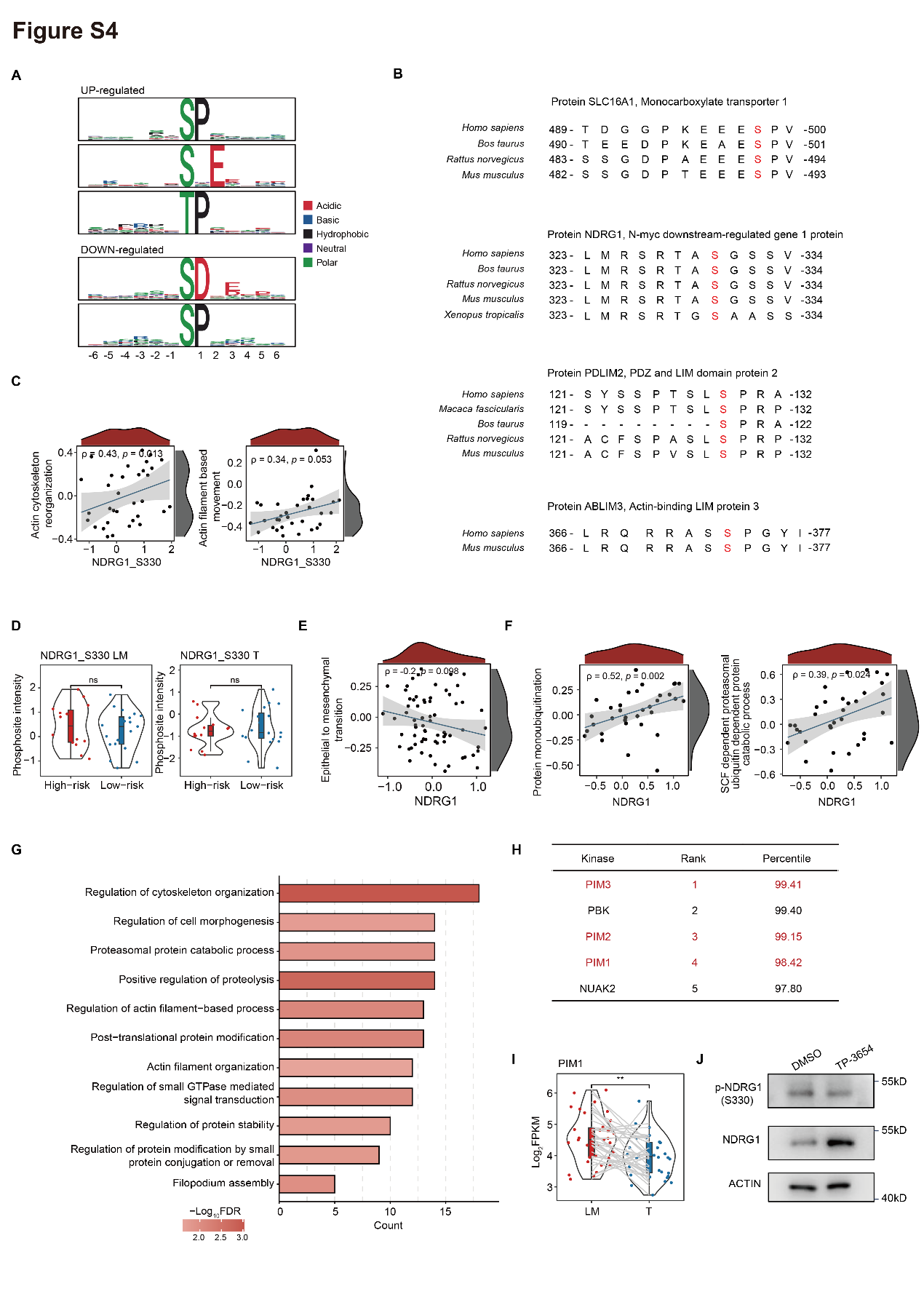
**

**Fig.S4.** Phosphoproteomics suggested the roles of several phosphosites in CRC metastasis. (A) Motif analysis was carried out using MoMo software tool and visualized using ggseqlogo. (B) Sequence alignment of the phosphosites on SLC16A1, NDRG1, PDLIM2 and ABLIM3 among different species. (C) Spearman correlation analysis between Ser330 phosphorylation on NDRG1 and two GO biological processes. (D) Boxplot and violin plot showed the quantification level of Ser330 phosphorylation on NDRG1 using Wilcoxon rank sum and signed rank test in T and LM samples with high and low CRS. (E) Spearman correlation analysis between protein expression of NDRG1 and EMT. (F) Spearman correlation analysis between protein expression of NDRG1 and two GO biological processes. (G) GO BP enrichment analysis of NDRG1 interaction proteins from BioGRID database. (H) List of the top 5 predicted kinases based on a publicly available resource^3^. (I) Boxplot and violin plot showed the mRNA quantification of *PIM1* kinase in our datasets (n = 34, two-sided Wilcoxon rank sum and signed rank tests). (J) Western blotting analyses of phosphorylated NDRG1 level with or without 3 μM pan PIM inhibitor TP-3654 treatment for 1 h. Actin expressions were shown as loading control. ∗ represented P < 0.05, ∗∗ represented P < 0.01, ∗∗∗ represented P < 0.001 and ns represented not significant.

**
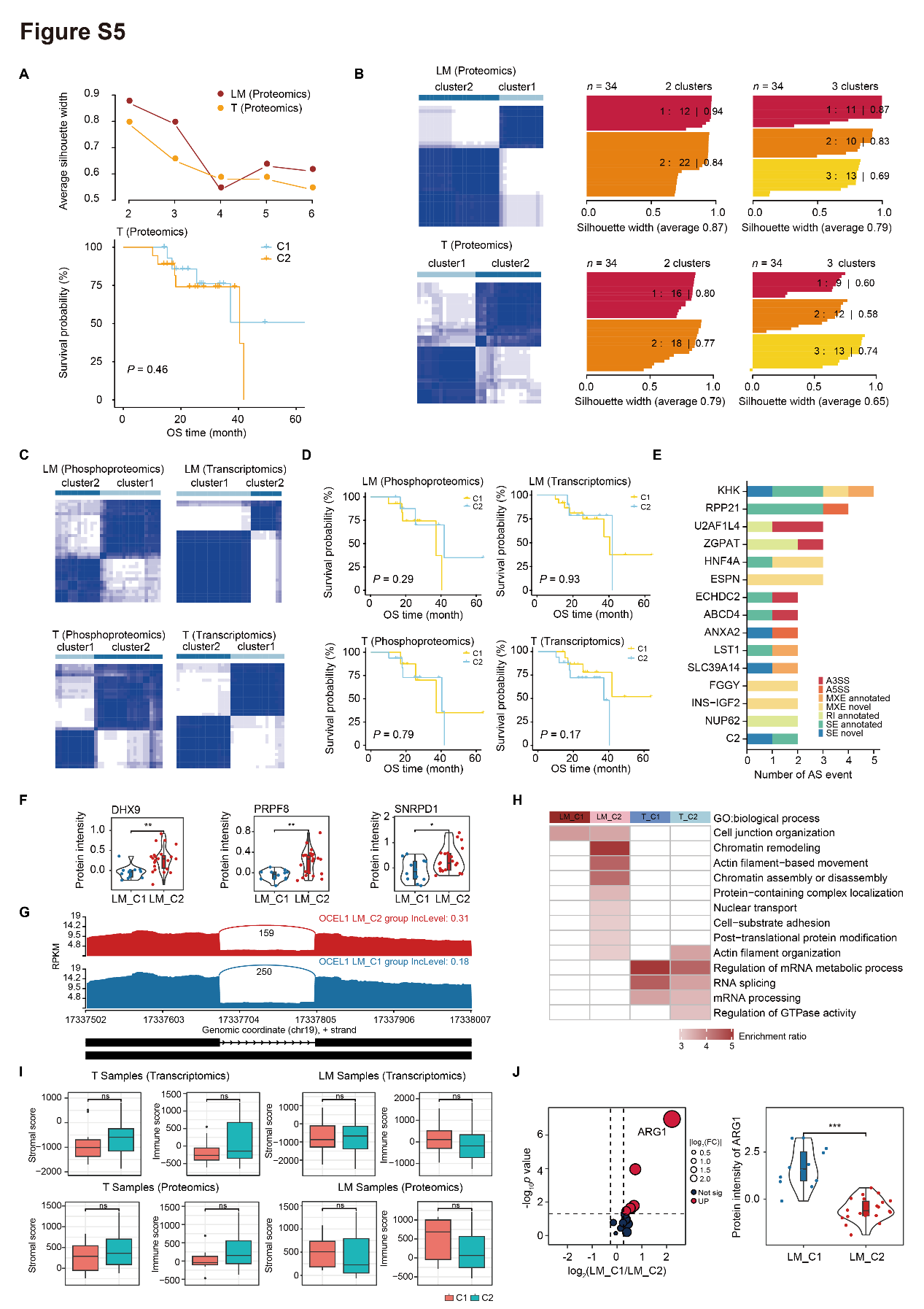
**

**Fig.S5.** Subtypes of CRLM samples. (A) Average silhouette width of unsupervised clustering with different k values based on proteomic data of LM and T samples (Top). Kaplan-Meier plot of OS for subsets based on T samples (Bottom). (B) Consensus matrix and silhouette plot of unsupervised clustering when k = 2 based on proteomic data of LM and T samples. (C) Consensus matrix of unsupervised clustering when k = 2 based on transcriptomic data and phosphoproteomic data using LM and T samples. (D) Kaplan-Meier plot of OS for subsets based on transcriptomic or phosphoproteomic data. (E) Genes with the top AS events that were significantly different between LM_C1 and LM_C2 samples. (F) Boxplot and violin plot showed the protein intensity of splicing-related factors in C1 and C2 (two-sided Wilcoxon rank sum and signed rank tests). (G) Sashimiplot of representative AS events between C1 and C2 subtype. (H) Phosphoproteomics indicated biological processes enriched in LM_C1, LM_C2, T_C1, and T_C2 groups (FDR < 0.05). (I) Boxplot of immune scores and stromal scores at transcriptomic level and proteomic level for C1 and C2 subtype patients, respectively (two-sided Wilcoxon rank sum and signed rank tests). (J) Volcano and violin plot of the protein expression of ARG1 between C1 and C2 subtype (two-sided Wilcoxon rank sum and signed rank tests). ∗ represented P < 0.05, ∗∗ represented P < 0.01, ∗∗∗ represented P < 0.001 and ns represented not significant.

**
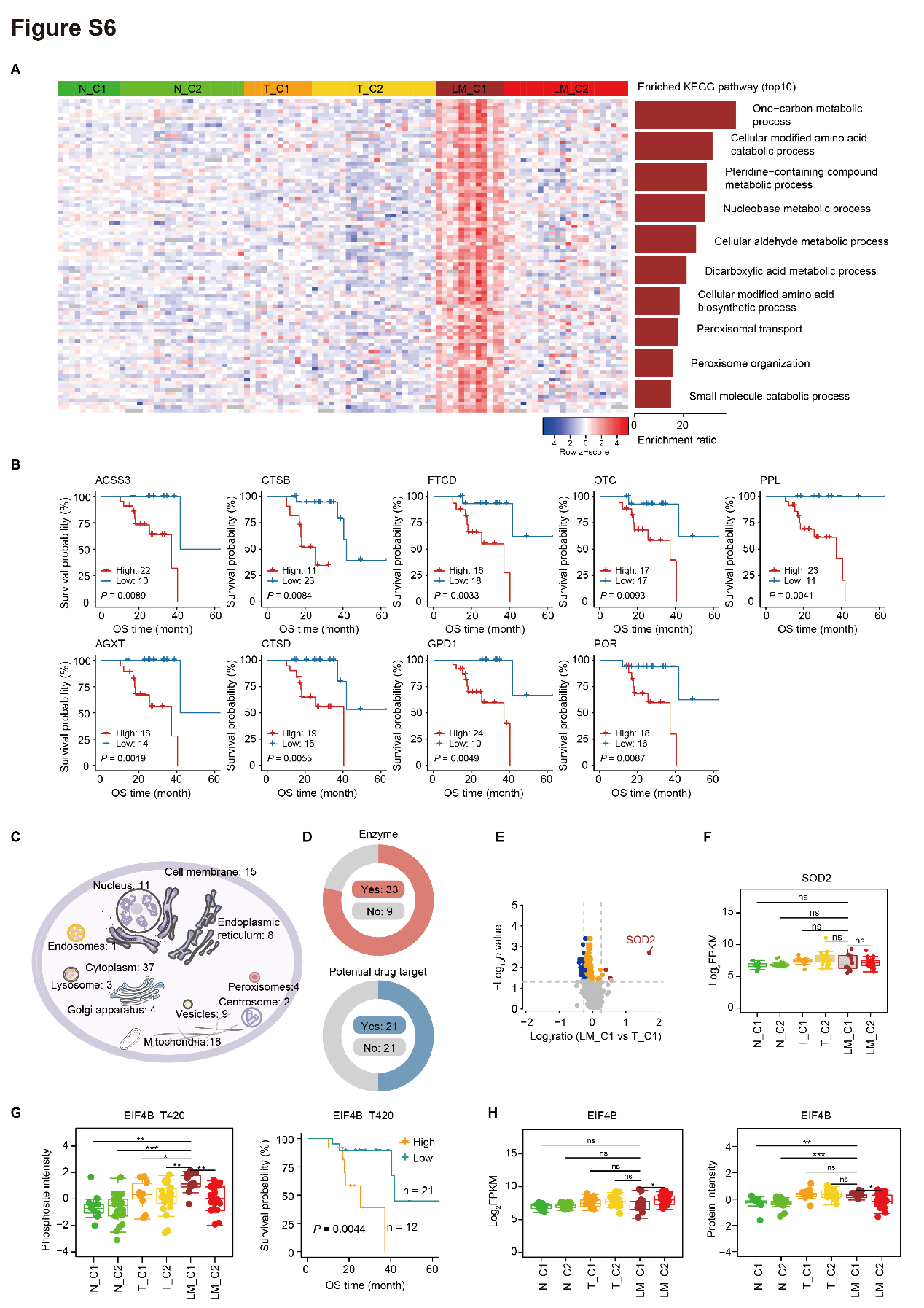
**

**Fig.S6.** Identification of potential protein and phosphosite biomarkers. (A) Heatmap of proteins highly expressed in LM_C1 subtype (two-sided Wilcoxon rank sum and signed rank tests) and KEGG pathway enrichment analysis results (right). (B) Kaplan-Meier plot of OS for the representative LM_C1 highly expressed proteins. (C) The subcellular location of the potential prognostic biomarkers (n = 42). (D) The proportion of potential prognostic biomarkers (n = 42) that could be classified as enzymes or potential drug targets. (E) Volcano plot presenting differentially expressed proteins between LM and T samples in C1 subtype patients (two-sided Wilcoxon rank sum and signed rank tests). (F) The mRNA level of SOD2 in C1 and C2 subtype patients of N, T, and LM samples (two-sided Wilcoxon rank sum and signed rank test). (G) The phosphorylation level of EIF4B_T420 among LM_C1, LM_C2, T_C1, T_C2, N_C1, and N_C2 groups (two-sided Wilcoxon rank sum and signed rank test) with clinical prognosis relevance (log-rank test, n = 33). Kaplan-Meier plot of OS stratified by EIF4B_T420 phosphorylation in the liver metastasis samples (n = 33). (H) Transcriptomic and proteomic level of EIF4B among LM_C1, LM_C2, T_C1, T_C2, N_C1, and N_C2 groups (two-sided Wilcoxon rank sum and signed rank test). ∗ represented P < 0.05, ∗∗ represented P < 0.01, ∗∗∗ represented P < 0.001 and ns represented not significant.

**Supplementary Methods**

**DNA extraction**

Total DNA were extracted using QIAamp DNA mini Kit (QIAGEN, Hilden, Germany) according to the manufacturer’s instructions. DNA degradation and contamination were monitored on 1% agarose gels. DNA concentration was measured by Qubit® DNA Assay Kit in Qubit® 2.0 Flurometer (Life Technologies, CA, USA). 0.6 μg extracted genomic DNA was used as input for DNA sequencing for each sample. Sequencing libraries were generated by using Agilent SureSelect Human All Exon V6 kit (Agilent Technologies, CA, USA) following manufacturer’s recommendations and barcodes were added to each sample. DNA fragments were sequenced on an Illumina NovaSeq 6000.

**Alignment**

The Trim_Galore script was used for adaptor trimming and low-quality reads filtering. Clean reads were then aligned to the human reference genome using BWA MEM with default parameters. The alignment results were converted to bam files, sorted and indexed by Samtools, and then marked and removed duplicate reads by picard. The GATK’s best practice was utilized to ran local realignment and recalibration.

**Variant detection and annotations**

Mutect2 was utilized to detect SNVs and small indels (insertion/deletion). Mutect2 was performed in default setting by setting T or LM samples and matched N samples as input, and only mutations located in targeted area were evaluated. Human SNP database, COSMIC coding and noncoding mutation data were additionally used as reference inputs of Mutect2. Mutations that met the standard of quality control were finally annotated by Oncotator.

**Somatic copy number alteration**

Somatic copy number alteration (SCNA) was analyzed by following the GATK’s Best Practice. The resulted segment files were analyzed using GISTIC2 to identify significantly amplified or deleted regions across all clinical samples. To exclude false positives as much as possible, relatively stringent thresholds were used with the following parameters: -ta 0.5 -tb 0.5 -brlen 0.5 -conf 0.9. Other parameters were the same as default values.

**RNA extraction and sequencing**

RNA was extracted from samples using TRIzol reagent kit (Ambion, Invitrogen, USA) according to the manufacturer’s protocols. RNA concentration and integrity were then checked by Qubit2.0 Fluorometer (Thermo Fisher Scientific) and Agilent 2100 Bioanalyzer (Agilent, CA, USA). Samples with an RNA integrity number (RIN) greater than 6.0 were used in the study. 3 μg RNA was used as input for the RNA sample preparations for each sample. Sequencing libraries were generated using NEBNext® UltraTM RNA Library Prep Kit for Illumina® (NEB, USA) following manufacturer’s recommendations and index codes were added to mark sequences to each sample. Briefly, purified mRNA from total RNA was fragmentated using divalent cations under elevated temperature in NEBNext First Strand Synthesis Reaction Buffer (5X). First strand cDNA was synthesized using random hexamer primer and M-MuLV Reverse Transcriptase, followed by second-strand synthesis using DNA Polymerase I and RNase H. Remaining overhangs were digested into blunt ends by exonuclease/polymerase activities. Then 3’ ends of DNA fragments were adenylated, and NEBNext Adaptor with hairpin loop structure were ligated to prepare for hybridization. The library fragments were purified with AMPure XP system (Beckman Coulter, Beverly, USA). Adaptor-ligated cDNA was treated with USER Enzyme (3 μL; NEB) at 37 °C for 15 min and 95 °C for 5 min, followed by PCR amplification. At last, PCR products were purified (AMPure XP system) and library quality was assessed on the Agilent Bioanalyzer 2100 system. The index-coded samples were then clustered and the library preparations were sequenced on an Illumina Hiseq platform and 150 bp paired-end reads were generated.

**RNA-Seq data analysis**

RNA-Seq reads were adaptor trimmed and the data quality was assessed with the FastQC software before any data filtering criteria was applied. Reads were mapped to the human reference genome by HISAT2 software. For quantification purpose, featureCounts were used with the genome annotation file and the relative abundance of the gene was measured by FPKM (Fragments Per Kilobase of transcript per Million mapped reads).

**Alternative splicing events detection and quantitation**

The clean RNA-seq reads were mapped onto the human reference genome using STAR software, and rMATS was used for exon-centric splicing events identification and quantitation between any two groups of interest, applying default parameters. Five major categories of alternative splicing (AS) events including alternative 5′ splice site (A5SS), alternative 3′ splice site (A3SS), mutually exclusive exon (MXE), skipped exon (SE), and retained intron (RI) were detected and quantified by rMATS. Splicing events that met the following criteria were defined as significantly differential AS events: absolute value of IncLevelDifference > 0.1 and FDR < 0.05. rmats2sashimiplot was used for AS visualization.

**Protein extraction and tryptic digestion**

Tissue samples were washed with phosphate buffer saline (PBS) buffer for two times. Next, the samples were minced and lysed in an 8M Urea lysis buffer containing 100 mM NH_4_HCO_3_, along with protease and phosphatase inhibitors (Roche, Mannheim, Germany). After incubating on ice for 25 min, the samples were sonicated for 2 min (two seconds on and five seconds off) and then centrifuged at 21,130 g for 10 min to remove debris. The supernatant was transferred to a new tube. Proteins were reduced with 5 mM DTT at 56 °C for 30 min, alkylated with 15 mM IAA in the dark at room temperature for 30 min, and then quenched with 30 mM cysteine. After dilution with 100 mM NH₄HCO₃ (pH 8.0), samples were digested with trypsin at 1:50 (w/w) enzyme-to-substrate ratio for 16 h at 37 °C, followed by a second digestion at 1:100 (w/w) for 4 h. Finally, the peptides were desalted by a Sep-Pak C18 column (Waters, Milford, MA, USA).

**Tandem mass tag (TMT) labeling**

Ten-plex TMT reagents were utilized to label the desalted peptides according to the manufacturer’s instructions (Thermo Fisher Scientific, San Jose, CA, USA). Internal reference sample (IRS) was prepared by mixing 63 samples (N, T, and LM samples at equal amount). The IRS was labeled with TMT 126 reagent through multiple runs of sample analyses, and the rest channels contained 9 samples including 3 adjacent normal colorectal samples, 3 CRC samples and 3 liver metastasis samples. The labeling efficiency was checked with an EASY-nLC 1200 system coupled to a Q Exactive HF-X mass spectrometer (Thermo Fisher Scientific, San Jose, CA, USA). After checking labeling efficiency (TMT modification ratio > 98% for both lysine residue and peptide N-termini), the peptides labeled by different TMT reagents were combined with equal contribution, dried using SpeedVac, and desalted by Sep-Pak C18 cartridges.

**Peptide fractionation by high-pH HPLC**

For proteomics analysis, high-pH reversed phase HPLC with a Waters XBridge Prep C18 column (5 μm particles, 4.6 × 250 mm) was used to reduce sample complexity. The TMT labeled peptides were dissolved in mobile phase A (2% acetonitrile, ammonium hydroxide solution, pH = 10). Mobile phase B contained 98% acetonitrile and 2% mobile phase A. Tryptic peptides were loaded and separated with a 69 min gradient at a flow rate of 1.0 mL/min. The LC gradient started with an increase of solvent B to 5% in 2 min, 5% to 12% B for 8 min, followed by linear rise to 33% B in 57 min, 2 min to 95% B. Finally, the peptides were eluted and mixed into twenty fractions and dried by a SpeedVac for further experiments.

For phospho-proteomics analysis, mobile phase A contained 4.5 mM ammonium formate and 2% acetonitrile, pH 10.0. Mobile phase B contained 4.5 mM ammonium formate and 90% acetonitrile. The LC gradient started with a 100% solvent A for 7 min. Next, a linear increase of solvent B to 16% B in 6 min, then linearly increased to 40% B in 60 min, 4 min to 44% B, 5 min to 60% B. The flow rate was 1.0 mL/min. Eventually, the peptides were eluted and mixed into twelve fractions and dried by a SpeedVac for further experiments.

**The enrichment of phosphorylated peptides**

Phosphorylated peptides were enriched as described^4^. Briefly, microspheres and Ti(SO_4_)_2_ with the ratio of 1:50 were mixed and incubated at room temperature overnight. After centrifugation at 800 g for 5 min, the supernatant was discarded. Then, the obtained Ti^4+^ microspheres (Ti^4+^-IMAC) were washed with 0.1% trifluoroacetic acid (TFA) buffer for 6 times, 50% ACN/6% TFA/200 mM NaCl buffer once, and subsequent 0.1% TFA buffer for 3 times. The Ti^4+^-IMAC adsorbents were stored at 4 °C before use. The Ti^4+^-IMAC adsorbents were dispersed in 0.1% TFA and introduced into the tips. Afterwards, Ti^4+^-IMAC spin tips were washed by 0.1% TFA buffer and equilibrated with loading buffer containing 80% ACN and 6% TFA twice. The protein digests were dissolved in 65% ACN, 100 mM NaCl, and 6% TFA to a final concentration of 1 mg/mL, and loaded on the tips twice to perform the complete binding. Then, tips were washed with 200 mM NaCl, 50% ACN, and 6% TFA twice to reduce non-specific binding peptides. To remove the salt, tips were further washed with 30% ACN and 0.1% TFA. Finally, the phosphorylated peptides were eluted by 10% NH_3_·H_2_O and dried down using a SpeedVac. Before LC-MS/MS analysis, the eluted peptides were desalted by C18 Zip Tips (Millipore Corporation, Billerica, MA).

**LC-MS/MS for TMT labeled proteome analysis**

The proteome fractions were separated using an EASY-nLC 1200 system (Thermo Fisher Scientific, San Jose, CA, USA). The analytical column was homemade reverse-phase C18 column (21 cm × 75 μm column, ReproSil-Pur 120 C18-AQ, 1.9 μm particle size, 120 Å pore size, Dr. Maisch GmbH, Germany). The peptides were dissolved in mobile phase A (0.1% formic acid in 2% acetonitrile). After sample loading, peptides were eluted for 65 min, maintaining at a constant column flow of 300 nL/min. The gradient was set from 6% to 30% mobile phase B (0.1% formic acid in 90% acetonitrile) in 57 min, 30% to 45% mobile phase B in 4 min, then 45% to 80% mobile phase B in 4 min. MS analysis was performed using a Q Exactive HF-X mass spectrometer (Thermo Fisher Scientific, San Jose, CA, USA). The scan range of precursor ions were m/z 350-1550. The resolution of precursor spectra was 60,000 at m/z 200. The automatic gain control (AGC) was set to 3e6 and maximum injection time (MIT) was 45 ms. In MS/MS acquisition, the top twenty ions were fragmented by higher-energy collision dissociation (HCD) with the normalized collision energy (NCE) of 32%. Afterwards, the fragment ions were detected in the Orbitrap with a resolution of 45,000 at m/z 200. AGC was set to 1e5, and the MIT was 30 ms. Masses selected for MS/MS were isolated at a width of 0.8 m/z. The dynamic exclusion duration was 30 s.

**LC-MS/MS for TMT phosphoproteome analysis**

The phosphorylated peptides were separated using an EASY-nLC 1200 system (Thermo Fisher Scientific, San Jose, CA, USA). The analytical column was homemade reverse-phase C18 column (21 cm × 75 μm column, ReproSil-Pur 120 C18-AQ, 1.9 μm particle size, 120 Å pore size, Dr. Maisch GmbH, Germany). The peptides were dissolved in mobile phase A (0.1% formic acid in 2% acetonitrile). After sample loading, peptides were eluted with 105 min, maintaining at a constant column flow of 300 nL/min. The gradient was set from 2% to 10% mobile phase B (0.1% formic acid in 90% acetonitrile) in 42 min, 10% to 22% mobile phase B in 45 min, then 22% to 40% mobile phase B in 15 min, and finally 40% to 80% mobile phase B in 3 min. MS analysis was performed using a Q Exactive HF-X mass spectrometer (Thermo Fisher Scientific, San Jose, CA, USA). The scan range of precursor ions was m/z 350-1800. The resolution of precursor spectra was 60,000 at m/z 200. The automatic gain control (AGC) was set to 3e6 and maximum injection time (MIT) was 50 ms. In MS/MS acquisition, the top ten ions were fragmentized by HCD with the NCE of 32%. Afterwards, the fragment ions were detected in the Orbitrap with a resolution of 45,000 at m/z 200. AGC was set to 1e5, and the MIT was 105 ms. Masses selected for MS/MS were isolated at a width of 0.8 m/z. The dynamic exclusion duration was 20 s.

**Proteomic database search.**

All MS/MS spectra were analyzed using MaxQuant software (1.6.7.0) against the UniProt human database including 96,464 sequences (downloaded in September 2019). TMT 10-plex-based MS2 reporter ion quantification with a mass tolerance of 0.003 Da was selected. The precursor intensity fraction (PIF) filter was set at 0.75 to reduce the interference of precursor co-fragmentation. Enzyme specificity was set as trypsin/P. The maximum missed cleavages were set as two. Cysteine carbamidomethylation was included as fixed modification. For global proteomic data, methionine oxidation and protein N-term acetylation were set as variable modifications. For phosphoproteomic data, an additional phosphorylation (+79.9663 Da) on serine, threonine or tyrosine residues were added. Less than six modifications per peptide were required for each peptide. The tolerances of first search and main search for peptides were set at 20 ppm and 4.5 ppm, respectively. The FDR cutoff for protein level, PSM level and modified site level was set as 0.01. For each batch of TMT labeling data, the purities of TMT labeling channels were corrected in terms of the kit LOT number.

**MS data pre-processing**

The reverse or potential contaminants in protein and modification lists were removed. For proteomic data, the intensity was median normalized, and then relative abundance was calculated as the ratio of sample abundance to IRS abundance and then log_2_-ratio transformed. For phosphoproteomic data, phosphorylated sites with localization probability ≥ 0.75 were remained. For phosphosite data of human tissues, the intensity was normalized by corresponding median of proteomic sample, and then relative abundance was calculated as the ratio of sample abundance to IRS abundance and then log_2_-ratio transformed. The time batches were removed using the “removeBatchEffect” function from the 'limma' R package, and the data was normalized by z-score for each sample. After removing batch effect, the results showed negligible residual batch effects and clear separation among the N, T, and LM groups.

**Data imputation**

For unsupervised clustering, we imputed missing values in the preprocessed omics data (protein, phosphosite, and mRNA data of human tissue) using the KNN method from the R package “impute”. For the public proteome data^2^ used in gene set enrichment analysis (GSEA), the intensities from the same gene were first summed before imputation. Genes or phosphosites that were present in at least 80% of the samples were retained using the following imputation parameters: k = 3, rowmax = 0.2, colmax = 0.2.

**Unsupervised clustering**

For each omics data (proteomics, phosphoproteomics and transcriptomics), we selected the most variant features (median absolute deviation (MAD) > 1) as input for unsupervised clustering using the R package “ConsensusClusterPlus”^5^ with the following parameters: maxK = 10, reps = 1000, clusterAlg = “km”, pItem = 0.8, finalLinkage = “average”, pFeature = 0.8, distance = “euclidean”. The number of clusters was demonstrated by the clearest consensus matrix and the rapid decrease of average silhouette score from k = 2 to 3.

**LM or T enriched protein set selection and GSEA analysis**

For LM enriched protein set, proteins were filtered with following criteria: 1) Upregulated in LM samples compared with T samples with a significance (median of fold-change > 1.2 and Wilcoxon rank sum and signed rank tests, P < 0.05). 2) No significance (median of fold-change ≤ 1.2 or Wilcoxon rank sum and signed rank tests, P ≥ 0.05) between T samples and N samples.

For T enriched protein set, proteins were filtered with following criteria: 1) Upregulated in T samples compared with N samples with a significance (median of fold-change > 1.2 and Wilcoxon rank sum and signed rank tests, P < 0.05). 2) Upregulated in T samples compared with LM samples with a significance (median of fold-change > 1.2 and Wilcoxon rank sum and signed rank tests, P < 0.05).

For the concise version, we additionally required the gene to be up- or down-regulated in more than 45% of samples ( ≥ 15).

For GSEA, the permutation type was set as “gene-set”. The metric for ranking genes was set to “Signal2Noise”.

**Gene set variation analysis (GSVA)**

We removed proteins with missing values more than 20% in all samples and imputed missing values utilizing KNN method as before. Normalized proteome matrix (log_2_ transformed ratio) was used for Gene Set Variation Analysis (GSVA) enrichment scores estimation via R package GSVA. KEGG pathway and GO biological process database were downloaded from GSEA MSigDB website (v2022.1). Minimum size of the overlap gene sets of each pathway gene list and query gene list was set as 5.

**Pathway analysis**

For transcriptomic and phosphoproteomic data, the genes (median of fold-change > 1.5 and Wilcoxon rank sum and signed rank tests, P < 0.05) showed significant difference. For proteomic data, the genes (median of fold-change > 1.2 and Wilcoxon rank sum and signed rank tests, P < 0.05) showed significance. These significantly differently expressed genes were further used for overrepresentation enrichment analysis (ORA) using WebGestalt website (<http://www.webgestalt.org/>) or R package clusterProfiler (v4.2.10)^6^. Significantly regulated pathways were required with a significance of FDR < 0.05.

**Principal component analysis (PCA)**

For mRNA, protein and phosphosite data, genes or phosphosites with no missing values were used for PCA analysis. The most variant proteins (median absolute deviation > 1) were used for PCA analysis. PCA analysis was conducted by R (v3.6.0) tools “FactoMineR” (v2.3) and “factoextra” (v1.0.7).

**Kinase-substrate enrichment analysis (KSEA)**

KSEA was performed by KESA App website (https://casecpb.shinyapps.io/ksea/)^7^ using phosphosite data according to its manual with “PhosphoSitePlus^8^ + NetworKIN^9^” dataset. The cutoff was set to P < 0.05 and substrate count ≥ 5.

**Protein-protein interaction (PPI) network analysis**

NDRG1 interaction proteins were derived from BioGRID database (v4.4.221) with Organism ID set as 9606^10^.

**Potential functionally important phosphorylation analysis**

Phosphosites with the same gene and phosphorylation site were combined by the median of log_2_ ratio. “ID mapping” tool of UniProt website (https://www.uniprot.org/id-mapping) was used for gene symbol mapping to the UniProt ID of public phosphosite function score annotation^11^. The following criteria were used: 1) Phosphosites with functional score > 0.5. 2) Phosphosites with a significance (median of fold-change > 1.5 and Wilcoxon rank sum and signed rank tests, P < 0.05) between different groups (LM and T samples, C1 and C2 subtypes).

**Prognostic relevance analysis**

The “survminer” R package was used to determine the optimal cutpoint for continuous variables using the maximally selected rank statistics from the 'maxstat' R package. Function “survfit” of “survival” R package was used to calculate the log-rank P value. The proteins or phosphosites with log-rank P < 0.05 were considered be prognosis relevant.

**Cancer dependent protein analysis**

The cancer-dependent genes were analyzed using genetic dependency of CRISPR screening dataset from DepMap database (https://depmap.org/portal/download/). The average of gene dependency was calculated for CRC and hepatocellular carcinoma cell lines, respectively. Genes with average of gene dependency < -0.6 were considered as potential CRC and hepatocellular carcinoma dependent genes.

**Motif analysis**

Motif analysis was carried out using MoMo software tool^12^ and visualized using ggseqlogo^13^.

**Animal model experiment**

All mice were maintained in a specific-pathogen-free (SPF) facility, and all related procedures were performed in compliance with the Guide for the Care and Use of Laboratory Animals were approved by the Institutional biomedical research ethics committee of Shanghai Institute of Nutrition and Health, Chinese Academy of Sciences (approval numbers: SINH-2021-QJ-2, SINH-2022-QJ-2, SINH-2023-QJ-2, SINH-2024-QJ-2; Shanghai, China). For cecal injection, 4 to 6-week-old mice were injected with 2×10^6^ HCT116 cells. For the liver metastasis model, 5×10^5^ KAP cells (derived from *Villin*^CreERT2^, *Kras*^LSL-G12D^, *Apc*^min/+^, *Trp53*^flox/flox^ mice following two months of tamoxifen treatment, mimicking common genetic alterations in CRC patients) were injected into the spleen. Bioluminescence imaging (BLI) was acquired with *in vivo* imaging systems (IVIS) Spectrum CT (PerkinElmer). For formate treatment, mice received water containing 125 mM of sodium formate (Sigma, 71539) two days before KAP cell injection, and sodium formate were administered every day. For AICAR treatment, mice received intraperitoneal injection of 50 mg/kg AICAR (Selleck, S1802) every other day after KAP cell injection. For formate assay experiment, tumors from mice livers were cut into proper weight and then measured using the Formate Assay Kit (Abcam, ab111748).

**Cell culture**

Human 293T Cell lines were purchased from Cell Bank, Shanghai Institute of Biochemistry and Cell Biology (SIBCB), Chinese Academy of Sciences. 293T cells were cultured in DMEM media (Gibco, C11965500BT) supplemented with 10% FBS (Gibco, 10270-106) and penicillin/streptomycin (Gibco, 15140122) at 37 °C under 5% CO_2_. Mouse KAP cells were derived from *Villin*^CreERT2^, *Kras*^LSL-G12D^, *Apc*^min/+^, *Trp53*^flox/flox^ mice following two months of tamoxifen treatment, mimicking common genetic alterations in CRC patients. KAP cells were embedded in Matrigel (Corning, 354248) and cultured in Advanced Dulbecco’s modified Eagle’s medium/F12 (Gibco, 12634028) supplemented with 10 mM HEPES (Gibco, 15630080), 2 mM GlutaMAX (Gibco, 35050061), penicillin/streptomycin, 1 mM N-acetylcysteine (Sigma, 112422) and 10 mM nicotinamide (Sigma, N0636) at 37 °C under 5% CO_2_. For treatments, KAP cells were cultured with 1 mM formate, 1mM glycine (MCE, HY-Y0966), 1mM methionine (MCE, HY-13694), 20 μM thymidine (MCE, HY-N1150), 20 μM purine (MCE, HY-34431), 20 μM NADPH (MCE, HY-F0003), 1 mM AICAR (MCE, HY-13417) or 3 μM TP-3654 (MCE, HY-101126).

**Expression plasmids, shRNA, sgRNA, CRISPRa and CRISPRi**

The full-length human *SHMT1*, *CPS1*, *NDRG1* and *NDRG1^S330A^* cDNA were cloned into pLVX-IRES-puro (Clontech) to generate expression plasmids. The *Shmt1 and Ndrg1* sense and antisense oligonucleotides were annealed and cloned into pLKO.1-Puro or pLKO.1-Neo (Addgene). pLentiCRISPRv2-sgRNAs targeting *Cps1* gene locus were used to knock out the gene in cells. sgRNAs for CRISPRko were designed by CRISPick (https://portals.broadinstitute.org/gppx/crispick/public). The shRNA and sgRNA sequences are listed in Supplementary Table 1.

**Transwell assay**

KAP cells (8 × 10^4^ cells/well) were seeded in the top chamber of the transwell (Corning, 353097). Media with or without 3 μM TP-3654 were added to the bottom layer of the transwell. After 18 h incubation, cells that migrated to the bottom chamber were counted.

**WB, IP and IB analysis**

For Western Blotting, protein was extracted by with SDS-loading and analyzed by western blotting.

For IP assays, cells were lysed and washed in HEPES lysis buffer (20 mM HEPES, pH 7.4, 200 mM NaCl, 1.5 mM MgCl2, 2 mM EGTA, 0.5% NP-40, 1 mM NaF, 1 mM Na_3_VO_4_ and 1 mM PMSF) supplemented with protease-inhibitor cocktail (Roche). Cell lysates were incubated overnight at 4 °C with indicated primary antibody and protein A/G agarose beads (Roche). Beads were centrifuged at 1000 g for 5 min at 4 °C to remove the supernatant, washed four times with the IP buffer and boiled SDS-loading buffer for 10 min at 95 °C. Samples were run on SDS-PAGE gel analyzed by western blotting. The following antibodies were used: anti-Beta Actin (Proteintech, 66009-1-Ig), anti-HA (Proteintech, 51064-2-AP), anti-NDRG1 (ABclonal, A4050), anti-Phospho-NDRG1-S330 (ABclonal, AP0807), Anti-Phospho-AMPKα (Thr172) (CST, 2535S), Anti-AMPKα (CST, 2532S), and Anti-Ubiquitin (CST, 3933S).

**Independent validation cohort of CRLM patients and tissue microarray (TMA) construction**

The independent validation cohort included 87 treatment-naïve CRLM patients treated in FUSCC. The TMA of liver metastasis samples of included patients were constructed as previously described in detail ^14^. Briefly, formalin-fixed, paraffin-embedded tissue blocks from resected liver metastasis were obtained. Tissue cylinders with a 0.6 mm diameter were punched from representative tissue areas of each donor tissue block and brought into one recipient paraffin block (30 × 25 mm). Each TMA spot included at least 50% tumor cells. For semi-quantification of immunohistochemical data, the quantified method is based on a multiplicative index of the average staining intensity (0 to 3) and extent of staining (0 to 3) in the cores, yielding a staining index ranging from 0 (no staining) to 9 (extensive, strong staining) ^15, 16, 17^. All the analyses were conducted or confirmed by two certified clinical pathologists independently. The staining score of ≤ 4 was defined as low expression, whereas the score of >4 was referred as high expression. The histological types were confirmed by experienced pathologists. The usage of clinical specimens was also approved by the Ethical Committee and Institutional Review Board of FUSCC. The clinical parameters including gender, age, primary site, histological subtype, TNM staging, differentiation, tumor size and prognostic information were enclosed into Supplementary Table 1, respectively. Immunohistochemical staining was used for validation and the following antibodies were used: anti-SHMT1 (Proteintech, 14149-1-AP), anti-GPD1 (Santa cruz, sc-376219), anti-FTCD1 (Santa cruz, sc-53128), anti-SOD2 (Proteintech, 24127-1-AP) and anti-EIF4B (Ser422) (Abcam, ab59300).

**Immunostaining**

For immunohistochemical staining, in brief, tissues or organoids were fixed in 4% paraformaldehyde (PFA) (Sigma, P6148) and embedded by paraffin and sectioned. Antigen retrieval was performed by boiling slides in citrate solution (Vector Laboratories) for 15 min microwave. Slides were permeated by 0.2% Tritox-X100, blocked with 5% goat serum, incubated with primary antibody at 4 ℃ overnight and peroxidase-conjugated secondary antibodies for 1 hour. The streptavidin-biotin ABC peroxidase immunohistochemistry kit (Vector Laboratories) was used to amplify signal and the antigens were stained by 3, 3’-diaminobenzidine (DAB). For immunofluorescence, after incubation with peroxidase-conjugated secondary antibodies, slides were incubated with dyes-labeled tyramide (Invitrogen) using the following primary antibodies, phospho-histone H3 (pH3) (Cell Signaling Technology, 53348) and cleaved-caspase 3 (CC3) (Cell signaling Technology, 9664s).

**List of abbreviations**

A3SS, alternative 3’ splice site; A5SS, alternative 5’ splice site; GO, gene ontology; BP, biological process; CAG, cancer-associated genes; CRC, colorectal cancer; CRLM, colorectal liver metastasis; CRS, clinical risk score; ECM, extracellular matrix; EMT, epithelial-to-mesenchymal transition; GSEA, gene set enrichment analysis; KSEA, Kinase substrate enrichment analysis; LM, liver metastasis tissues; mCRC, metastatic CRC; MEX, mutually exclusive exons; N, adjacent normal colorectal tissues; OS, overall survival; PCA, principal component analysis; PFS, progression-free survival; RI, retained introns; SCNVs, somatic copy-number variations; SE, skipped exons; T, CRC tissues; TME, tumor microenvironment; WES, whole exome sequencing.

**Supplementary Reference**

1. Gillette, M.A., Satpathy, S., Cao, S. et al. Proteogenomic Characterization Reveals Therapeutic Vulnerabilities in Lung Adenocarcinoma. Cell. 2020;182:200-225.e235.

2. Li, C., Sun, Y.D., Yu, G.Y. et al. Integrated Omics of Metastatic Colorectal Cancer. Cancer cell. 2020;38:734-747.e739.

3. Johnson, J.L., Yaron, T.M., Huntsman, E.M. et al. An atlas of substrate specificities for the human serine/threonine kinome. Nature. 2023;613:759-766.

4. Yao, Y., Dong, J., Dong, M. et al. An immobilized titanium (IV) ion affinity chromatography adsorbent for solid phase extraction of phosphopeptides for phosphoproteome analysis. Journal of Chromatography A. 2017;1498:22-28.

5. Wilkerson, M.D. & Hayes, D.N. ConsensusClusterPlus: a class discovery tool with confidence assessments and item tracking. Bioinformatics. 2010;26:1572-1573.

6. Xu, S., Hu, E., Cai, Y. et al. Using clusterProfiler to characterize multiomics data. Nat Protoc. 2024;19:3292-3320.

7. Wiredja, D.D., Koyuturk, M. & Chance, M.R. The KSEA App: a web-based tool for kinase activity inference from quantitative phosphoproteomics. Bioinformatics. 2017.

8. Hornbeck, P.V., Zhang, B., Murray, B. et al. PhosphoSitePlus, 2014: mutations, PTMs and recalibrations. Nucleic Acids Res. 2015;43:D512-520.

9. Horn, H., Schoof, E.M., Kim, J. et al. KinomeXplorer: an integrated platform for kinome biology studies. Nat Methods. 2014;11:603-604.

10. Stark, C., Breitkreutz, B.J., Reguly, T. et al. BioGRID: a general repository for interaction datasets. Nucleic Acids Res. 2006;34:D535-539.

11. Ochoa, D., Jarnuczak, A.F., Viéitez, C. et al. The functional landscape of the human phosphoproteome. Nature Biotechnology. 2020;38:365-373.

12. Cheng, A., Grant, C.E., Noble, W.S. et al. MoMo: discovery of statistically significant post-translational modification motifs. Bioinformatics. 2019;35:2774-2782.

13. Wagih, O. ggseqlogo: a versatile R package for drawing sequence logos. Bioinformatics. 2017;33:3645-3647.

14. Sauter, G., Simon, R. & Hillan, K. Tissue microarrays in drug discovery. Nat Rev Drug Discov. 2003;2:962-972.

15. Huang, Y., Hu, K., Zhang, S. et al. S6K1 phosphorylation-dependent degradation of Mxi1 by β-Trcp ubiquitin ligase promotes Myc activation and radioresistance in lung cancer. Theranostics. 2018;8:1286-1300.

16. Liu, J., Zhang, C., Zhao, Y. et al. Parkin targets HIF-1α for ubiquitination and degradation to inhibit breast tumor progression. Nature communications. 2017;8:1823.

17. Yuan, H., Han, Y., Wang, X. et al. SETD2 Restricts Prostate Cancer Metastasis by Integrating EZH2 and AMPK Signaling Pathways. Cancer cell. 2020;38:350-365.e357.
